# Personalized Cardiac Computational Models: From Clinical Data to Simulation of Infarct-Related Ventricular Tachycardia

**DOI:** 10.3389/fphys.2019.00580

**Published:** 2019-05-15

**Authors:** Alejandro Lopez-Perez, Rafael Sebastian, M. Izquierdo, Ricardo Ruiz, Martin Bishop, Jose M. Ferrero

**Affiliations:** ^1^Center for Research and Innovation in Bioengineering (Ci2B), Universitat Politècnica de València, Valencia, Spain; ^2^Computational Multiscale Simulation Lab (CoMMLab), Universitat de València, Valencia, Spain; ^3^INCLIVA Health Research Institute, Valencia, Spain; ^4^Arrhythmia Unit, Cardiology Department, Hospital Clínico Universitario de Valencia, Valencia, Spain; ^5^Division of Imaging Sciences & Biomedical Engineering, Department of Biomedical Engineering, King's College London, London, United Kingdom

**Keywords:** myocardial infarction (MI), ventricular tachycardia (VT), border zone (BZ), electrical remodeling (ER), fibrosis, slow conducting channel (SCC), computational simulation, radiofrequency ablation (RFA)

## Abstract

In the chronic stage of myocardial infarction, a significant number of patients develop life-threatening ventricular tachycardias (VT) due to the arrhythmogenic nature of the remodeled myocardium. Radiofrequency ablation (RFA) is a common procedure to isolate reentry pathways across the infarct scar that are responsible for VT. Unfortunately, this strategy show relatively low success rates; up to 50% of patients experience recurrent VT after the procedure. In the last decade, intensive research in the field of computational cardiac electrophysiology (EP) has demonstrated the ability of three-dimensional (3D) cardiac computational models to perform *in-silico* EP studies. However, the personalization and modeling of certain key components remain challenging, particularly in the case of the infarct border zone (BZ). In this study, we used a clinical dataset from a patient with a history of infarct-related VT to build an image-based 3D ventricular model aimed at computational simulation of cardiac EP, including detailed *patient-specific* cardiac anatomy and infarct scar geometry. We modeled the BZ in eight different ways by combining the presence or absence of electrical remodeling with four different levels of image-based patchy fibrosis (0, 10, 20, and 30%). A 3D torso model was also constructed to compute the ECG. *Patient-specific* sinus activation patterns were simulated and validated against the patient's ECG. Subsequently, the pacing protocol used to induce reentrant VTs in the EP laboratory was reproduced *in-silico*. The clinical VT was induced with different versions of the model and from different pacing points, thus identifying the slow conducting channel responsible for such VT. Finally, the real patient's ECG recorded during VT episodes was used to validate our simulation results and to assess different strategies to model the BZ. Our study showed that reduced conduction velocities and heterogeneity in action potential duration in the BZ are the main factors in promoting reentrant activity. Either electrical remodeling or fibrosis in a degree of at least 30% in the BZ were required to initiate VT. Moreover, this proof-of-concept study confirms the feasibility of developing 3D computational models for cardiac EP able to reproduce cardiac activation in sinus rhythm and during VT, using exclusively non-invasive clinical data.

## Introduction

Cardiovascular disease is the leading cause of both morbidity and mortality worldwide, with ischemic heart disease being the most common cause among them (Nowbar et al., 2014; Abubakar et al., 2015). Months or even years after suffering from a myocardial infarction (MI), when the healing process is complete and the MI has reached the chronic stage (van den Borne et al., 2010; Daskalopoulos et al., 2012), a significant number of patients develop potentially lethal ventricular tachycardias (VT) due to the arrhythmogenic nature of remodeled tissue (Lazzara and Scherlag, 1984; de Bakker et al., 1993; Nguyen et al., 2014). Infarct-related VTs are commonly associated with the so-called *slow conducting channels* (SCC), also known as *isthmuses*, which are pathways composed of surviving myocytes across the infarct scar that are responsible for the initiation and maintenance of reentrant activity, usually leading to monomorphic VTs (de Bakker et al., 1988; Aliot et al., 2009). Moreover, SCC are closely related to the *border zone* (BZ) (also termed *gray zone* or *peri-infarct zone*), a region constituting the transition between infarct scar and healthy myocardium that comprises altered but still viable tissue surrounding the dense fibrotic scar (infarct scar) resulted from the healing of MI. Several studies have described the BZ as a region of slowed conduction composed of surviving but remodeled myocytes with infiltration of bundles of patchy fibrosis extending from the core of compact fibrosis (infarct scar), which results in a highly arrhythmogenic tissue (de Bakker et al., 1993; Rohr, 2012; Rutherford et al., 2012;Nguyen et al., 2014).

At present, cardiac delayed enhancement-MRI (DE-MRI) is commonly used to explore the infarcted area pre-operatively, since it enables *in-vivo* evaluation of the tissue damaged by MI (i.e., scar and BZ) due to the hyper-enhancement of the infarcted region in the images (Kim et al., 1999a; Fieno et al., 2000; Doltra et al., 2013). In fact, it is currently considered as the gold-standard test for *in-vivo* assessment of scar and myocardial viability after MI in clinical settings (Jamiel et al., 2017; Patel et al., 2017). Cardiac DE-MRI provides a substrate characterization after MI that has shown close correlation with histopathological analyzes (Kim et al., 1999a; Fieno et al., 2000; Wagner et al., 2003; Amado et al., 2004), allowing to differentiate between scar and BZ. The usefulness of MRI-based substrate characterization and SCCs delineation for planning and guiding ablation procedures aimed at infarct-related VTs has been tested in numerous studies (Ashikaga et al., 2007; Andreu et al., 2011, 2015, 2017; Perez-David et al., 2011; Wijnmaalen et al., 2011; Fernández-Armenta et al., 2013; Soto-Iglesias et al., 2016; Yamashita et al., 2016).

Radiofrequency ablation (RFA) is a common procedure to interrupt reentrant circuits through SCC responsible for VTs related to chronic MI (Stevenson et al., 1993; de Chillou et al., 2002; Wilber, 2008; Berruezo et al., 2015; Baldinger et al., 2016). As part of the electrophysiological (EP) study immediately prior to RFA in patients with infarct-related VT, interventional cardiologists try to induce the clinical VT originally undergone by the patient by means of pacing protocols applied at selected sites of myocardium. A positive induction of monomorphic VT is assumed as an evidence of the presence of at least one SCC responsible for the VT (Pedersen et al., 2014; Priori et al., 2015). In such a case, the SCC is ablated to block the propagation through the reentrant circuit, thus avoiding the reentry and, consequently, the VT. However, these procedures are invasive, risky and very time-consuming. Moreover, they show a relatively low success rate, as up to 50% of patients develop recurrent VT after the RFA procedure (Gerstenfeld, 2013; Yokokawa et al., 2013; Baldinger et al., 2016).

Electroanatomical mapping (EAM) systems (Ben-Haim et al., 1996; Gepstein et al., 1997), are commonly employed in the EP laboratory to guide RFA procedures aimed at assessing both atrial (Calkins et al., 2012) and ventricular arrhythmias (Aliot et al., 2009; Priori et al., 2015), due to its ability to integrate spatial 3D and EP information recorded by the catheter. In the case of infarct-related VTs, EAM systems are considered as a helpful tool to identify SCCs as RFA targets based on the abnormal features of electrograms (EGM) in such regions (Gardner et al., 1985; Bogun et al., 2005), especially when clinical VT is unmappable due to non-inducibility or hemodynamic instability (Marchlinski et al., 2000; Aliot et al., 2009; Priori et al., 2015).

In contrast to EAM systems, an alternative non-invasive approach for pre-operative characterization of target substrate and planning of RFA procedures is the use of 3D computational models able to simulate cardiac EP accurately. Such an approach has the potential to help physicians to better understand and predict the underlying mechanisms of a wide variety of cardiac disorders, as well as in therapy planning and management of those diseases (Vigmond et al., 2009; Smith et al., 2011; Trayanova et al., 2012, 2017; Krueger et al., 2013; Trayanova and Boyle, 2013; Lopez-Perez et al., 2015; Arevalo et al., 2016). Although such *in-silico* approaches show significant potential, a number of important questions related to the necessary level of detail required by the model remain unanswered. In particular, the specific manner in which the microscopic structural and functional remodeling, known to be present in the infarct BZ, is represented in these macroscopic clinical models, and the subsequent implications of these specific choices in the initiation and the sustenance of reentrant VT events, has yet to be fully investigated. Related to this, the degree of model personalization necessary for accurate simulation of *patient-specific* VT episodes is also a matter of debate; for example, the requirement of only personalized anatomy (from images), or whether the inclusion of functional personalization (from ECG or EAM data, for instance) should also be an essential feature in model construction. Full exploration of these effects is often limited by clinical data availability.

The main goal of this project is to develop a pipeline for performing personalized *in-silico* EP studies able to aid electrophysiologists in surgical planning of RFA procedures aimed at infarct-related VTs. For this purpose, we first evaluate the feasibility of building image-based 3D *patient-specific* models of ventricles and torso of chronically infarcted patients, as well as the capability of such models to reproduce patients' cardiac EP during VT episodes by computational simulation, including simulated ECGs aiming to compare them with patients' clinical recordings. Here we present a feasibility case study, building computational 3D models of ventricles and torso that are exclusively based on non-invasive high-resolution clinical data. The ventricular model includes personalized and detailed 3D geometry of both cardiac anatomy and the heterogeneous remodeling resulting from the MI healing process (infarct scar and BZ), since both factors seem to be essential to identify SCCs as RFA targets by means of *in-silico* EP studies. Additionally, as part of the EP modeling associated with our pipeline, we explore the impact on VT inducibility and VT features of different modeling strategies for the BZ, in which we include electrical remodeling (ER) and structural remodeling in the form of different densities of image-based patchy fibrosis in order to assess the arrhythmogenic effects of those factors, both separately and in combination.

## Materials and Methods

### Clinical Data

In this work, we used a set of clinical data from a 58-year-old male patient referred for a RFA procedure aimed at finishing a monomorphic VT related to a large chronic MI, which extended over seven out of the 17 segments of the left ventricle (LV) model of the American Heart Association (AHA). Myocardial regions that appear affected in the DE-MRI were basal and medial segments of both inferoseptal and inferolateral walls and all segments of the inferior wall (basal, mid, and apical), which correlates with an occlusion of the right coronary artery (Ortiz-Pérez et al., 2008). The patient suffered the acute MI 11 years before the clinical VT episode related to the MI. The clinical dataset includes: clinical high-resolution cardiac DE-MRI, whole thorax MRI, EAMs recorded via CARTO system (Biosense Webster, Inc., Diamond Bar, CA, USA) (Gepstein et al., 1997) and 12-lead ECG signals recorded during the RFA procedure both in sinus rhythm and during VT episodes induced by pacing. Importantly, all those data were not specifically generated for research purposes, but they were collected in a clinical environment as part of its daily routine.

Cardiac DE-MRI was acquired by a MRI scanner Magnetom Avanto 1.5T (Siemens Healthcare, Erlangen, Germany) using a phased-array body surface coil, about 15 min after the administration of the gadolinium-based contrast MultiHance (gadobenate dimeglumine, 529 mg/ml) (Bracco Diagnostics Inc., Monroe Township, New Jersey, USA). The acquisition was synchronized with both ECG (ECG-gated) and breathing (navigator-gated), imaging the heart at the end-diastolic phase of cardiac cycle (trigger delay = 685 ms, for a nominal R-R interval of 928 ms along the acquisition). The DE-MRI stack comprised 96 slices of 256 × 256 pixels encompassing the whole heart (ventricles and atria), with a pixel size of 1.4 × 1.4 mm and a slice thickness of 1.4 mm, thus resulting in isotropic voxel.

*In vivo* EP data were recorded by CARTO 3 system using the *NaviStar ThermoCool* ablation catheter with 3.5 mm saline-irrigated tip (Abdelwahab and Sapp, 2007). A pressure sensor placed at catheter tip ensured the proper contact between the catheter and the myocardial wall. A total of 847 points were registered: 315 from LV endocardium, 78 from right ventricle (RV) endocardium and 454 from epicardium.

Regarding the ethical considerations, the protocol was approved by the *Ethics Committee for Clinical Research of the Hospital Clinic Universitari de Valencia* (Valencia, Spain), which certifies that the present study was conducted in accordance with the recommendations gathered in the Declaration of Helsinki, originally adopted by the General Assembly of the World Medical Association in 1964, and in its subsequent revisions. Furthermore, the patient, who underwent the standard clinical protocol, gave written informed consent for the use of his anonymized clinical data in this study.

### 3D *Patient-Specific* Ventricular Model

#### Anatomical Model

**Figure 1:**
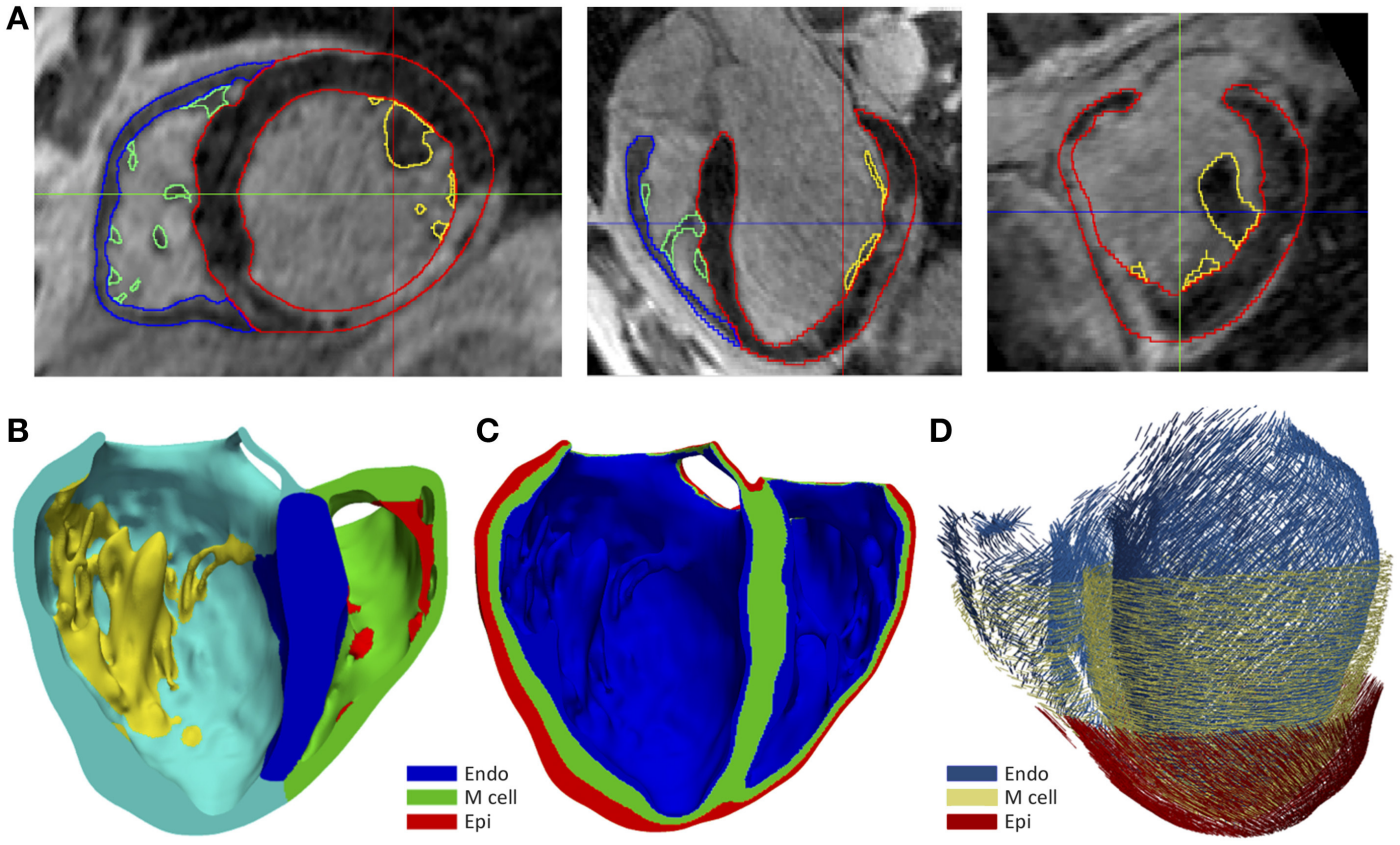
**(A)** Manual segmentation process of cardiac DE-MRI performed with Seg3D, displaying three slices corresponding to the standard cardiac planes: short-axis (*left*), four-chamber plane (*center*) and long-axis showing the LV (*right*). (Note that in those views the LV appears on the right side and the RV on the left). Contours highlighted in distinct colors show: LV myocardium including the septum (*red*), RV myocardium (*blue*) and papillary muscles and endocardial trabeculations of LV (*yellow*) and RV (*green*). **(B)** Posterior view of a coronal cross-section (four-chamber plane) of the hexahedra-based FEM volume mesh of the 3D *patient-specific* ventricular model. Various cardiac regions are labeled with different colors: septum (*blue*), LV free wall (*cyan*), RV free wall (*green*) and papillary muscles and main endocardial trabeculations of LV (*yellow*) and RV (*red*). **(C)** Transmural heterogeneity of ventricular myocardium, showing the distribution of the three different kinds of ventricular myocytes: endo-, mid- (M cells), and epicardial cells. **(D)** Left antero-lateral view of the 3D ventricular model displaying a representation of cardiac fibers orientation at different wall depths (endocardium, mid-myocardium, and epicardium).

We generated the 3D *patient-specific* bi-ventricular model by segmenting the short-axis slices from the cardiac DE-MRI using Seg3D software (Scientific Computing and Imaging Institute, University of Utah, USA) (Seg3D, 2013). We did it manually to perform a highly detailed segmentation of whole ventricles, including papillary muscles and main endocardial trabeculations (see Figures 1A,B). An expert radiologist in cardiac imaging checked all segmentations in order to ensure the fidelity of the 3D reconstruction of the *patient-specific* anatomy. From the segmented DE-MRI stack, we generated a surface model of ventricles that we carefully checked with Blender (Blender Foundation, Amsterdam, The Netherlands) to refine and correct defects in the mesh at the local level after applying a global smoothing. Then, using the surface model as a template, we performed a volume meshing with MeshGems-Hexa (Distene S.A.S., Bruyeres-le-Chatel, France), obtaining a hexahedra-based volume mesh comprised by 4 million nodes (vertices) and 3.71 million elements, with an average edge length of 0.4 mm.

To include the anisotropy of the cardiac muscle, we implemented Streeter's rule-based method (Streeter et al., 1969) modeled by the set of equations described in Sebastian et al. (2009), defining the helix (α_*h*_) and transmural (α_*t*_) angles (see Figure 1D) for every hexahedral mesh element, based on the apex-base relative distance and transmural depth of its position within the 3D model. We first applied this method to the whole LV myocardium (including septum) and then to RV free wall. In papillary muscles and endocardial trabeculations, fibers are known to be aligned parallel to the longitudinal axis of those anatomical structures (Greenbaum et al., 1981). In order to reproduce such configuration, we performed the topological skeletonization of the volume mesh to extract the medial axes of each one of those structures, what enabled to properly assign the fiber orientation within them. Finally, we performed a Gaussian smoothing with a 3D kernel to soften abrupt transitions in fibers direction between the myocardial wall and the papillary muscles and trabeculations, as well as at the RV free wall-septum junctions.

#### Infarct Scar and Border Zone

**Figure 2:**
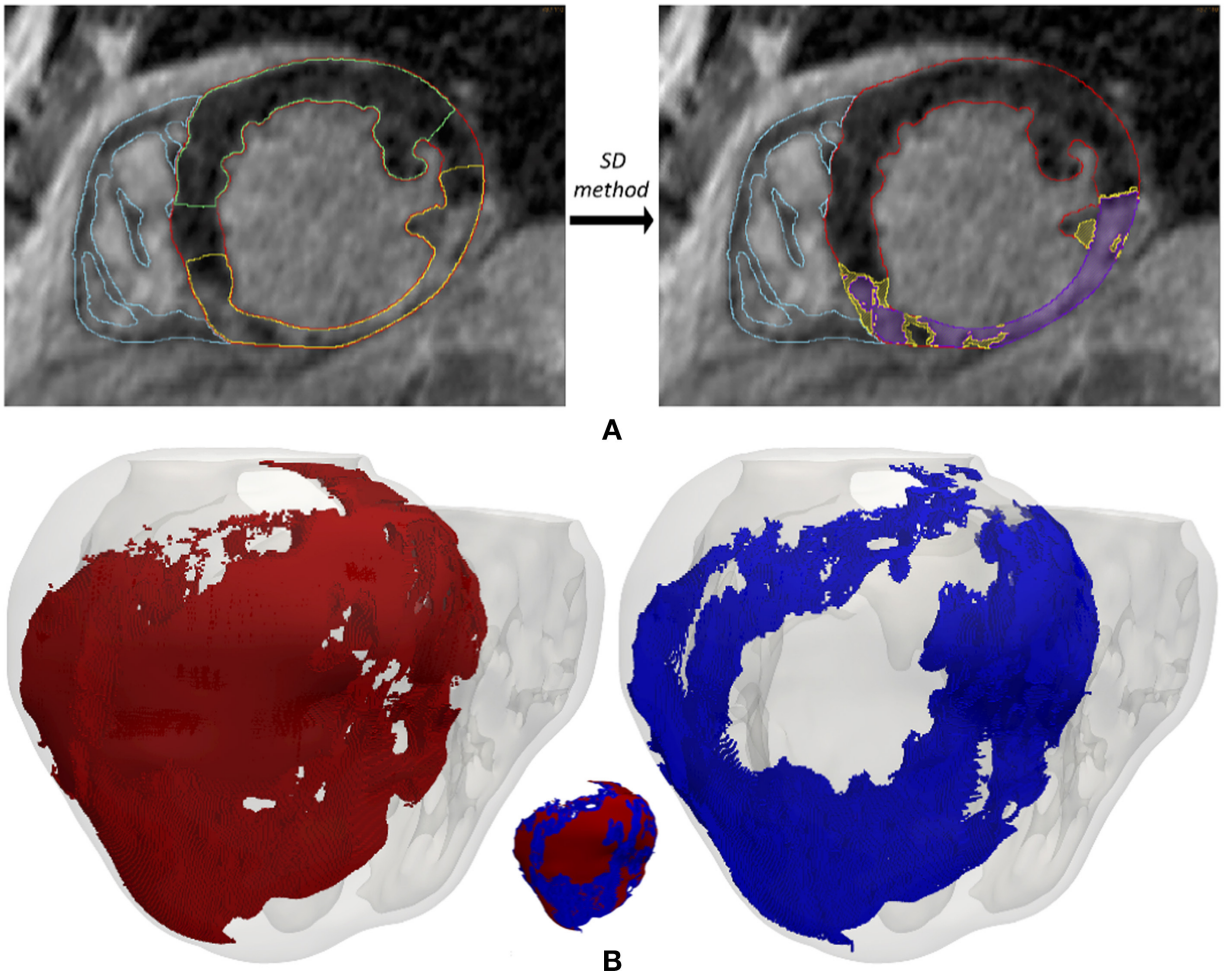
**(A)** Segmentation process from short-axis DE-MRI slices of the heterogeneous remodeling caused by MI, including infarct scar and BZ. Left panel shows the first step that involves the manual delineation of remote ROI (region-of-interest) (*green*) and infarcted ROI (*yellow*) within the LV myocardium ROI (*red*), also displaying the RV myocardium ROI (*cyan*). Right panel shows the segmentation for infarct scar (*purple*) and BZ (*yellow*) resulting from the application within the infarcted ROI of thresholds determined by SD method from the remote ROI of each slice. **(B)** 3D representation of the MI reconstructed from DE-MRI images, showing posterior views of the 3D surface model of ventricles (rendered with transparency) along with those hexahedral elements of volume mesh labeled as infarct scar (*red*) and BZ (*blue*).

We generated 3D representations of the geometry of the infarct scar and BZ by segmenting the slices of the cardiac DE-MRI by means of a custom implementation of the so-called *standard deviation* (SD) *method* (Kim et al., 1999a). Briefly, we divided the segmented LV myocardium into two sub-regions (healthy and infarcted) (see Figure 2A), based on the pixel intensity levels (i.e., gray levels). Within the infarcted region, we classified every pixel as dense fibrotic scar (intensity levels higher than mean+3 × SD of healthy myocardium), BZ (levels between mean+2 × SD and mean+3 × SD) or healthy tissue (values under mean+2 × SD) (Kim et al., 1999a; Fieno et al., 2000; Kolipaka et al., 2005; Yan, 2006; Perez-David et al., 2011). Finally, we mapped the scar and BZ into the volume mesh of the 3D ventricular model, labeling every hexahedron in the 3D model as healthy, scar or BZ depending on its position relative to the surfaces representing the infarct scar and the whole MI (see Figure 2B).

**Figure 3:**
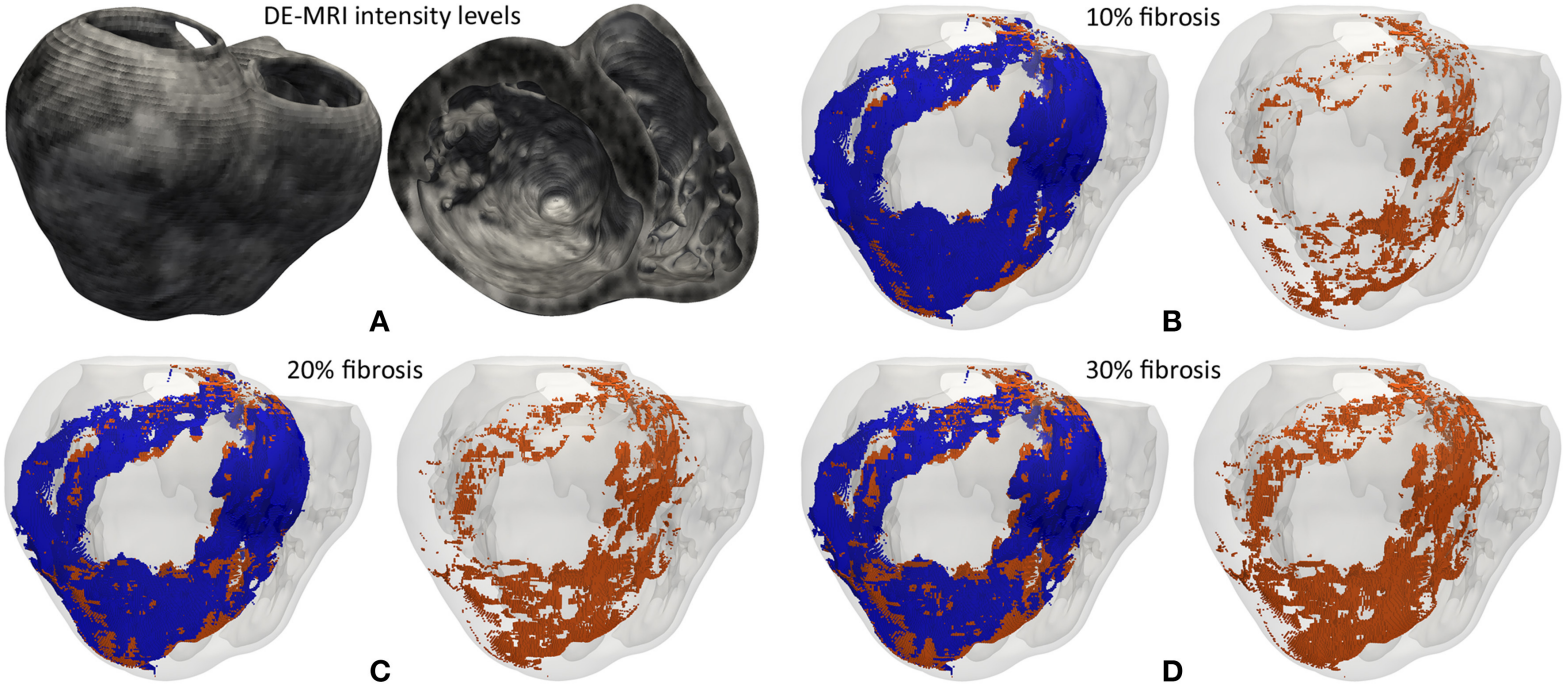
**(A)** Gray intensity levels from DE-MRI mapped into the hexahedral volume mesh of the 3D ventricular model, showing a postero-basal view of the whole model (*left*) and a basal view of a cross-section in the short-axis plane (*right*). The next three panels show the 3D fibrosis distribution resulting from the introduction of several degrees of structural remodeling within the BZ: **(B)** 10%, **(C)** 20%, and **(D)** 30% patchy fibrosis. All those panels show a posterior view of the 3D surface model of ventricles (rendered with transparency) together with those hexahedral elements labeled as BZ (*blue*) and fibrosis (*orange*) extracted from the volume mesh of ventricular model. In each panel there is an image showing both the BZ and fibrosis (*left*) and another one only displaying the fibrotic elements (*right*).

In an attempt to reproduce the structural remodeling within the BZ (patchy fibrosis) based on our clinical data, we first mapped the intensity levels of the DE-MRI into the volume mesh of our 3D ventricular model (Figure 3A). Since the DE-MRI voxels (isotropic voxels with edge length of 1.4 mm) were larger than FEM mesh elements (hexahedra with average edge length of 0.4 mm), the intensity level corresponding to each DE-MRI voxel was assigned to several FEM elements that might comprise up to 64 hexahedra. We then generated different levels of fibrosis (10, 20, and 30%) within the BZ based on that information mapped from the DE-MRI (see Figure 3). Among the elements of the volume mesh previously labeled as BZ, we defined as fibrotic those ones associated with the highest intensity levels, in an amount depending on the desired fibrosis level. Thus, we labeled as fibrotic a percentage of elements belonging to the BZ that matched the specified level of patchy fibrosis.

As shown in Figure 3, our approach of image-based generation of different fibrosis levels within the BZ resulted in clusters of fibrotic tissue. This method produced a distribution that may be classified as patchy fibrosis, defined as a mixture of bundles of myocardial tissue and fibrotic tissue (de Jong et al., 2011).

#### CARTO Data Reannotation and Integration

Local activation times (LAT) annotations were automatically determined by the *Confidense* module of CARTO system. First, we discarded all points with peak-to-peak amplitude under 0.5 mV in distal bipolar EGM (M1-M2), as they are considered as non-excitable tissue corresponding to the dense fibrotic scar (Marchlinski et al., 2000; Soejima et al., 2002). For the remaining EGMs, those acquired on healthy tissue were revised by a custom code for LAT re-annotation. We chose the deflection of distal bipolar signal closest to the point of maximum negative slope in distal unipolar signal as the activation time (Spach et al., 1979; Paul et al., 1990; Stevenson and Soejima, 2005), which showed good agreement with annotations determined by the *Confidense* module in most cases. However, such criterion is not reliable for points showing noisy and fragmented signals, typically located at the BZ surrounding the infarct scar (Aliot et al., 2009). In those cases, we placed LAT annotations manually under the close supervision of a well-trained electrophysiologist, who also reviewed LAT annotations for healthy EGMs. Furthermore, we discarded those points whose LAT value showed a poor spatio-temporal coherence with their closest neighbors. After this process, only 385 out of the 847 CARTO measurement points were preserved: 84 for LV endocardium, 49 for RV endocardium and 252 for epicardium.

**Figure 4:**
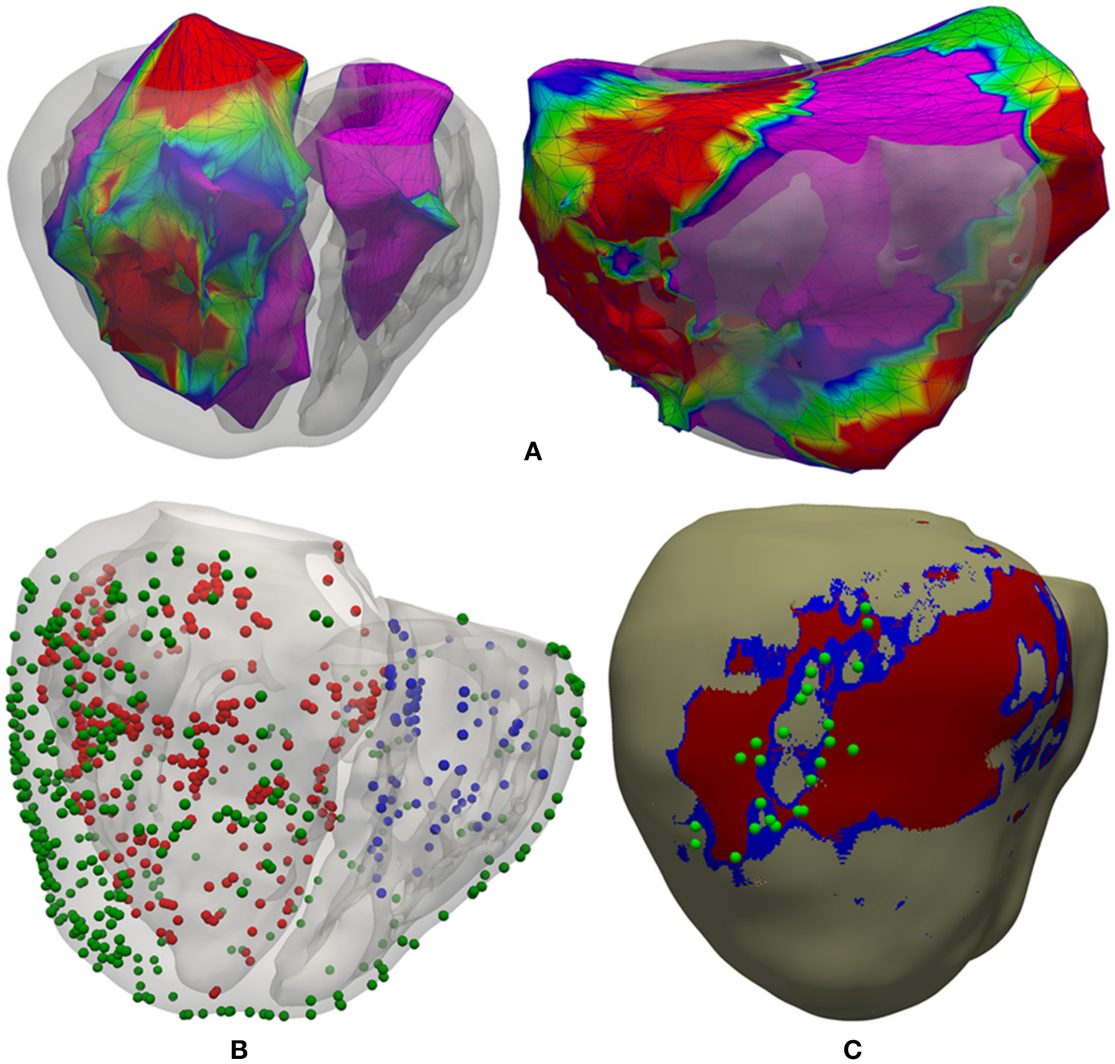
Registration process between CARTO surface meshes and the 3D ventricular model. **(A)** Two posterior views of the 3D surface model of ventricles rendered with transparency, showing registered CARTO surfaces for both endocardia (*left*) and epicardium (*right*). **(B)** CARTO measurement points projected onto the 3D model with colored spheres representing CARTO points recorded from LV endocardium (*red*), RV endocardium (*blue*), and epicardium (*green*). **(C)** Postero-lateral view of the 3D ventricular model showing a possible epicardial SCC mainly composed of BZ (*blue*) intermingled with some thin patches of healthy tissue at epicardial level (*beige*) and completely surrounded by infarct scar (*red*), closely matching the location of projected CARTO points tagged during the RFA procedure as candidates to be part of a SCC (*green spheres*) according to the features of their EGMs.

We had three surfaces generated by the CARTO system, including both acquired and interpolated data: LV endocardium, RV endocardium and epicardium (Figure 4A). Epicardial mapping was performed by accessing pericardial space through percutaneous (non-surgical) transthoracic subxiphoid approach by means of puncture using an epidural needle (Brugada et al., 2003; Sosa and Scanavacca, 2005; Tedrow and Stevenson, 2009; Yamada, 2014), following the access procedure originally described by Sosa et al. (1996, 2000). We applied a rigid transformation using the ICP (iterative closest point) algorithm (Besl and McKay, 1992), to align those three CARTO surface meshes to our 3D *patient-specific* ventricular model. Then, we projected all measurement points from each CARTO mesh to the closest surface node of our ventricular model, in terms of Euclidean distance. Thus, we obtained all CARTO measurement points (those preserved after the checking process) mapped onto the external surface of our 3D bi-ventricular model (see Figure 4B). The earliest activated region was located on the septal region of the LV endocardium at mid-apical level and, the latest activated on the epicardial surface within the BZ near the scar.

### 3D Torso Model

**Figure 5:**
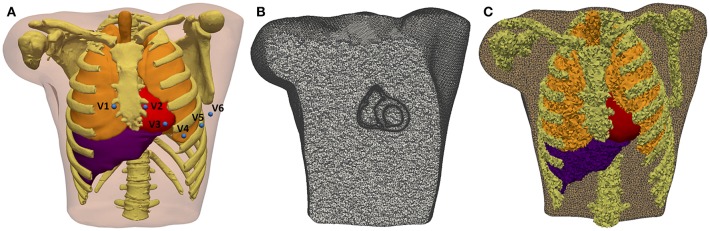
3D Torso model. **(A)** Anterior view of the torso model, showing the 3D surfaces that represents all organs and tissues included (bones, lungs, liver, ventricles, atria, and blood pools of four cardiac chambers). Blue spheres represent the location of virtual electrodes corresponding to precordial leads for ECG computation, placed in its standard positions. **(B)** Coronal cross-section of volume mesh of the 3D torso model, showing the edges of tetrahedral elements that are highly refined in the region of ventricles. **(C)** Labeled volume mesh. Different colors indicate to which organ or anatomical structure each tetrahedral element belongs as a result of the labeling process of volume mesh: lungs (*orange*), bones (*yellow*), liver (*purple*), and ventricles (*red*).

The whole torso MRI stack was acquired in the coronal plane with a slice thickness of 10 mm. We roughly segmented the main organs (lungs, liver, heart) and structures (bones, body contour, blood pools, great vessels) using Seg3D software. The resolution of the torso MRI hampered a detailed reconstruction of some important structures, so we used the reconstructed parts of the model as landmarks to fit a detailed torso model previously developed (Ferrer et al., 2015) by means of a linear transformation. Next, we replaced the ventricles in the fitted detailed torso model by our *patient-specific* model and removed any intersections between our ventricular model and surrounding organs (see Figure 5A). Finally, we used TetGen (Si and Gärtner, 2005) to mesh the torso volume with tetrahedra, which resulted in 1.26 million nodes and 7.38 million elements organs (see Figure 5B). The average edge length was of 0.55 mm. Note that the problem of passive propagation of extracellular potentials, i.e., only diffusion without reaction component, does not require such a fine spatial resolution outside the heart domain (Prassl et al., 2009); for this reason, the mesh is highly refined only in the region of the ventricles, as shown in Figure 5B.

### Electrophysiological Modeling

#### Healthy Myocardium

For healthy myocardium, we used the ten Tusscher model of human ventricular action potential (AP) (ten Tusscher and Panfilov, 2006), considering the transmural heterogeneity of the ventricular myocardium (Drouin et al., 1995; Antzelevitch et al., 1999). We defined three different transmural layers for endocardial, mid myocardial (M cells) and epicardial myocytes within the volume mesh of our ventricular model, spanning 17, 41, and 42% of ventricular wall thickness, respectively (see Figure 1C). These values were estimated from the data reported by several experimental studies (Sicouri and Antzelevitch, 1991; Sicouri et al., 1994; Yan et al., 1998).

In order to establish the conductivities that will define the conduction velocities (CV), we performed a set of test simulations on a 3D slab model (20 × 20 × 6 mm) composed of regular hexahedral elements (voxels) with an edge length of 0.4 mm, matching the average length in the ventricular model. As a result, we set the conductivity values to 0.24 S/m and 0.0456 S/m for longitudinal (σ_*L*_) and transversal (σ_*T*_) conductivity, respectively. This resulted in a CV of 0.68 m/s along the fiber direction and of 0.26 m/s in transverse direction. These values are consistent with the experimental measurements in human ventricles (Taggart et al., 2000).

#### Infarct Scar and Border Zone

We considered the infarct scar as a non-excitable tissue mainly composed of collagen, so we modeled it as an electrical insulator. To do so, we defined an internal boundary in our 3D model and imposed no-flux boundary condition at the interface between the scar and surrounding tissue, which mostly corresponded to the BZ.

To include the ER in the BZ, we modified the ten Tusscher model (ten Tusscher and Panfilov, 2006) by introducing reductions in the maximum conductance of certain ionic channels, as reported from several patch-clamp experiments with cells of epicardial BZs from canine hearts. According to those data, I_Na_ was reduced to 38% of its normal value (Pu and Boyden, 1997), I_CaL_ to 31% (Dun et al., 2004) and I_Kr_ and I_Ks_ to 30 and 20% (Jiang et al., 2000), respectively. At the tissue level, we reduced the conductivity parameters in the BZ to 0.05 S/m and 0.01 S/m for longitudinal (σ_*L*_) and transversal (σ_*T*_) conductivity, respectively. Importantly, we used the same conductivity values for the BZ for those versions of the model including fibrosis and ER and for those ones including only fibrosis in the BZ. Hence, we set reduced conductivities at tissue level for the BZ in all versions of the ventricular model regardless of whether or not it included ER at the ionic level. CVs measured on a 3D slab model using the remodeled version of ten Tusscher model were 0.17 m/s in the longitudinal direction and 0.065 m/s in the transverse directions, corresponding to a reduction of around 75% with respect to the CVs in healthy tissue. Conductivities for BZ in combination with the original ten Tusscher model yielded CVs of 0.225 m/s and 0.09 m/s for longitudinal and transverse directions, respectively, which means a reduction of around 65%.

For fibrosis within the BZ, we used the MacCannell model for human ventricular fibroblast (MacCannell et al., 2007). At tissue level, we considered fibrotic tissue as isotropic and set its conductivity to 0.1 S/m; a reduction of 60% with respect to the longitudinal conductivity in healthy myocardium. Fibrotic tissue behaves as a passive conductor that only enables electrotonic conduction (passive propagation), rapidly leading to blocks due to the attenuation of propagated potentials. Since the conductivity value set for fibrosis affects the coupling between myocytes and fibroblasts, it defines the magnitude of the electrotonic load exerted by the fibroblasts over the adjacent myocytes (MacCannell et al., 2007), with fibroblasts acting as electrical sinks along plateau and repolarization phases, while behaving as electrical sources during resting state (Jacquemet, 2006; Miragoli et al., 2006; Xie et al., 2009; Rohr, 2012; Gomez et al., 2014; Trayanova et al., 2014; Mahoney et al., 2016; Zeigler et al., 2016). This electrotonic interaction between myocytes and fibroblasts can cause a considerable impact on electrical conduction at tissue and organ level depending on fibroblast density, as will be observed in our simulation results.

#### Electrical Modeling of the Torso

We automatically labeled every tetrahedral element of the volume mesh as belonging to a given organ. The 3D torso model included bones, lungs, liver, whole heart (ventricles and atria) and blood pools of all cardiac chambers organs (see Figure 5C). As in Ferrer et al. (2015), conductivity values assigned to different organs and tissues were taken from the literature (MacLeod et al., 1991; Gabriel et al., 1996; Klepfer et al., 1997; Bradley et al., 2000; Bressler and Ding, 2006). We considered isotropic propagation for all organs and tissues of our 3D torso model, except for the ventricular myocardium where we preserved the anisotropy imposed by the orientation of cardiac fibers. As in Klepfer et al. (1997), for the space not covered by any organ or anatomical structure we set a conductivity of 0.239 S/m calculated as the average of the conductivities for the other tissues, including the skeletal muscle that was not considered as a specific region in our torso model. Finally, to simulate ECG signals we defined virtual electrodes on the surface of torso model corresponding to the precordial leads, which were placed in their standard positions (see Figure 5A).

### Computational Simulations

#### Simulations at the Organ Level

All simulations at the organ level were performed on eight different versions of our 3D *patient-specific* ventricular model, generated by combining the presence or absence of ER in the BZ with four different levels of image-based patchy fibrosis (0, 10, 20, and 30%), as detailed in Table 1. Thereby, we could independently assess the arrhythmogenic effect of the structural remodeling (fibrosis) and ER within the BZ, as well as the combination of both factors. We also performed some additional simulations with the model with ER and 10% fibrosis (model *#6*), aiming to study the effect of changing the conductivities within the BZ. We tested two new sets of values (0.12–0.03 S/m and 0.22–0.0485 S/m for longitudinal-transversal conductivities), which in combination with our remodeled version of the ten Tusscher model, respectively, resulted in CVs in the BZ reduced by 50 and 25% with respect to the CVs for healthy tissue.

**Table 1:** Different versions of the computational model of ventricles according to the kind of remodeling included in the BZ (ER and/or fibrosis) and results of *in-silico* VT inducibility tests performed with CVs in BZ reduced by 75% with respect to the healthy tissue.

**Model version**	**Fibrosis in BZ (%)**	**ER in BZ**	**Pacing site**		
			***endo#1***	***epi#1***	***epi#2***
*#1*	0	NO	no VT	no VT	no VT
*#2*	10		no VT	no VT	no VT
*#3*	20		no VT	no VT	no VT
*#4*	30		no VT	S3−290 ms 510 ms−117 bpm	no VT
*#5*	0	YES	no VT	S2−360 ms 506 ms−118 bpm	no VT
*#6*	10		S3−370 ms 526 ms−114 bpm	S2−360 ms 526 ms−114 bpm	no VT
*#7*	20		S3−370 ms 520 ms−115 bpm	S2−360 ms 520 ms−115 bpm	no VT
*#8*	30		no VT	no VT	no VT

*In the cases of positive VT induction, we specify the premature stimulus (S2 or S3) responsible for the unidirectional block triggering the reentrant VT and its CI, as well as the BCL and heart rate associated with the VT*.

To perform the simulations at the organ level, we used the software ELVIRA (Heidenreich et al., 2010), a FEM solver specifically developed for solving the anisotropic reaction-diffusion equation of the monodomain model for cardiac EP (Roth, 1988). For the numerical solution of our simulations, we applied the conjugate gradient method with an integration time step of 0.02 ms, using implicit integration for the parabolic partial differential equation of monodomain model and explicit integration with adaptive time stepping for the systems of ordinary differential equations associated with the ionic models.

##### Stabilization of myocyte-fibroblast coupling

Adjacent myocytes and fibroblasts are known to interact by coupling and signaling between them in both healthy and diseased myocardium (Kohl and Gourdie, 2014; Mahoney et al., 2016; Ongstad and Kohl, 2016). Electrotonic interaction induces changes in myocytes coupled to fibroblasts, such as elevation of resting potential (i.e., less negative) and APD shortening as a consequence of the effect of fibroblasts as electrical sinks (MacCannell et al., 2007; Zeigler et al., 2016). Thus, we needed to stabilize the myocyte-fibroblast electrotonic couplings as a first step in our simulation pipeline to let those interactions reach the steady state. For all models including any fibrosis level (10, 20, or 30%), first we performed a 1 s simulation without any stimulus. After an initial stabilization that caused a multi-foci ectopic-like activation, myocyte-fibroblast couplings remained stable in absence of stimulation (see Video S1), so the final state of those simulations would later serve as a starting point for the following ones in our simulation pipeline.

#### Simulation of the ECG

To obtain ECG signals on the body surface, we used an approximation of the bidomain model (Geselowitz and Miller, 1983) to compute the extracellular potentials across the torso volume. This approximation, described elsewhere (Keller et al., 2010) and recently used in other works (Ferrer-Albero et al., 2017; Martinez-Mateu et al., 2018), comprises several steps. First, transmembrane potentials, previously computed by simulation at the organ level using the solver ELVIRA as explained above, were interpolated from the ventricular model to the nodes of torso model corresponding to the ventricular myocardium. Then, solving the passive term (only diffusion) of the bidomain approach we obtained the extracellular potentials in the ventricles from the interpolated transmembrane voltages. Finally, applying Dirichlet boundary conditions at the ventricles-torso interface and Neumann-type conditions at the torso surface, the extracellular potentials were computed by using the FEM method to solve a Laplace equation over the volume mesh of 3D torso model. To obtain the numerical solution of the problem, we used the conjugate gradient method with the incomplete Cholesky decomposition as a pre-conditioner. (See Supplementary Material for more details about the method used to compute simulated ECGs).

## Results

### 3D Ventricular Model

As well as including *patient-specific* cardiac anatomy, our 3D ventricular model also integrated personalized geometry of heterogeneous remodeling caused by MI, differentiating between infarct scar and BZ that comprised 16 and 8.5% of the volume of LV myocardium, respectively. Importantly, 3D reconstruction of the MI remodeling from DE-MRI revealed the presence, at the epicardial level, of an isthmus mainly composed of BZ intermingled with several thin patches of healthy tissue that was surrounded by dense scar. Both the shape and features of this structure match the definition of a SCC representing a potential substrate for reentrant VT (de Bakker et al., 1988; Fernández-Armenta et al., 2013). Moreover, as observed in Figure 4C, integration of CARTO data onto the 3D model showed good agreement between the epicardial isthmus and a set of CARTO points labeled during the RFA procedure as candidates to be part of a SCC due to the characteristics of its bipolar EGMs (low voltage, fractionated and split signals, late and isolated potentials, etc.) (de Chillou et al., 2002; Bogun et al., 2005). The image-based *patient-specific* 3D model of infarcted ventricles developed in this work, as well as the 3D torso model, are both publicly available at http://commlab.uv.es/repository/.

#### Electrophysiological Modeling of the Border Zone

**Figure 6:**
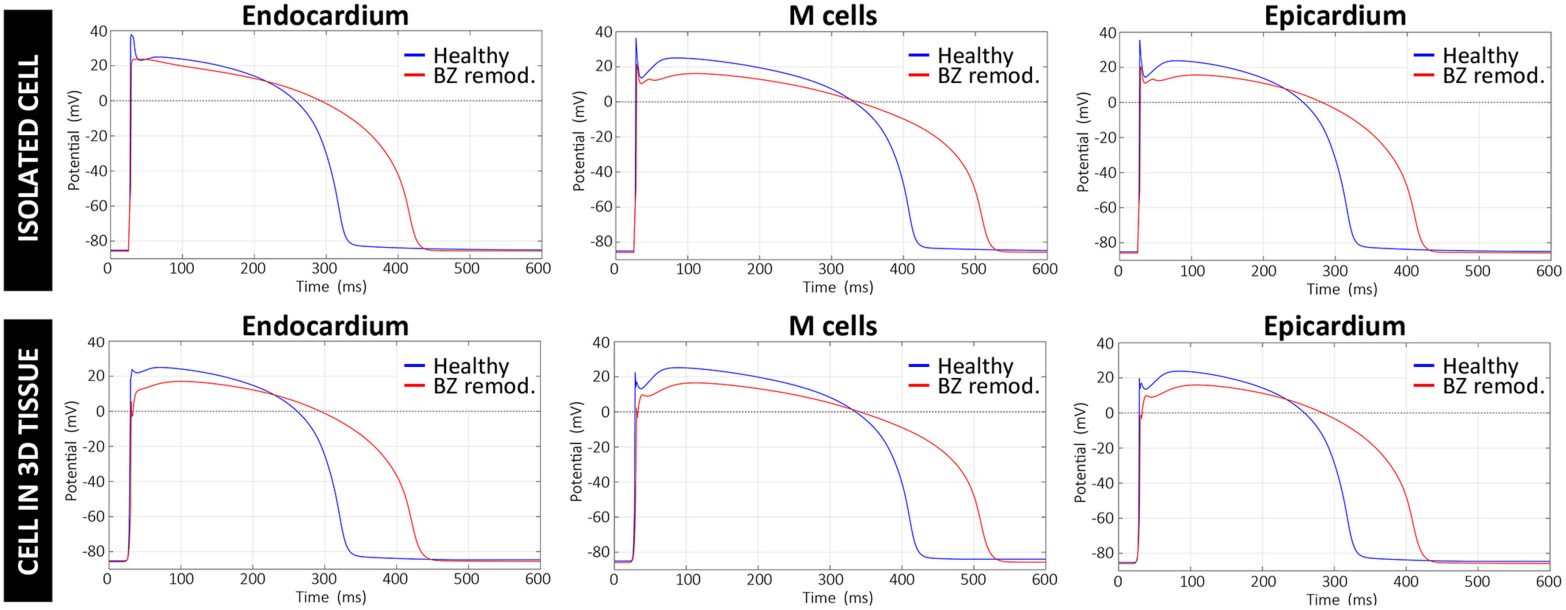
Comparison between APs generated by the original version of ten Tusscher model (ten Tusscher and Panfilov, 2006), used in our simulations for healthy myocardium, and APs resulting from our modified version of such model for the remodeled BZ. Upper row shows APs from a simulation with a single isolated cell, after stabilizing the model at a BCL of 800 ms, while the lower row displays APs obtained for a cell embedded in a 3D tissue (i.e., surrounded by other cells and electrically coupled to them) exclusively composed of remodeled myocytes.

ER in the BZ, represented by our modified version of ten Tusscher model, resulted in a decrease of upstroke velocity and maximum amplitude of AP mainly caused by the downregulation of I_Na_, as well as an increase in AP duration (APD) relative to the normal values due to the reduction of repolarizing potassium currents I_Kr_ and I_Ks_. As observed in Figure 6, the main effect of the applied changes is the prolongation of the APD, which affects both a single isolated cell and a cell embedded in a 3D remodeled tissue in a similar degree. Table S1 summarizes a quantitative analysis of the changes in several AP key biomarkers resulting from the modifications included in the ten Tusscher model for ER in the BZ, for both isolated and embedded cell.

### Computational Simulations

#### Sinus Rhythm Simulation

We reproduced the patient's ECG in sinus rhythm using computational simulation, in order to test and validate our 3D *patient-specific* model. Since we had the endocardial EAMs for both ventricles, we used that information to generate a *patient-specific* stimulation sequence aiming to reproduce the patient's sinus activation pattern accurately. We used a total of 133 manually-checked endocardial CARTO points (84 for LV and 49 for RV) mapped onto the endocardial surfaces to create that personalized sinus stimulation sequence. We used those mapped points as stimulation sites, applying a stimulus at each one of them in the time instant given by the checked LAT value associated with the corresponding endocardial CARTO point. The endocardial CARTO point with the earliest LAT was the first stimulated site (at *t* = 0 ms) and the rest of points were sequentially stimulated until reaching the latest activated point (highest LAT value) according to recorded EAMs (see Figure S1). To perform these simulations of sinus rhythm, we took the final state of the stabilization step (myocyte-fibroblast coupling) as starting point (*t* = 0 ms). Then, we applied the CARTO-derived endocardial stimulation sequence to simulate six heartbeats at a basic cycle length (BCL) of 800 ms, thus matching the patient's heart rate in sinus rhythm (75 bpm) during the RFA procedure, which was measured from the ECG recordings included in CARTO data. Note that only the sixth beat was used to simulate the ECG signals in the 3D torso model for a single heartbeat in order to compare those signals to patient's recordings.

**Figure 7:**
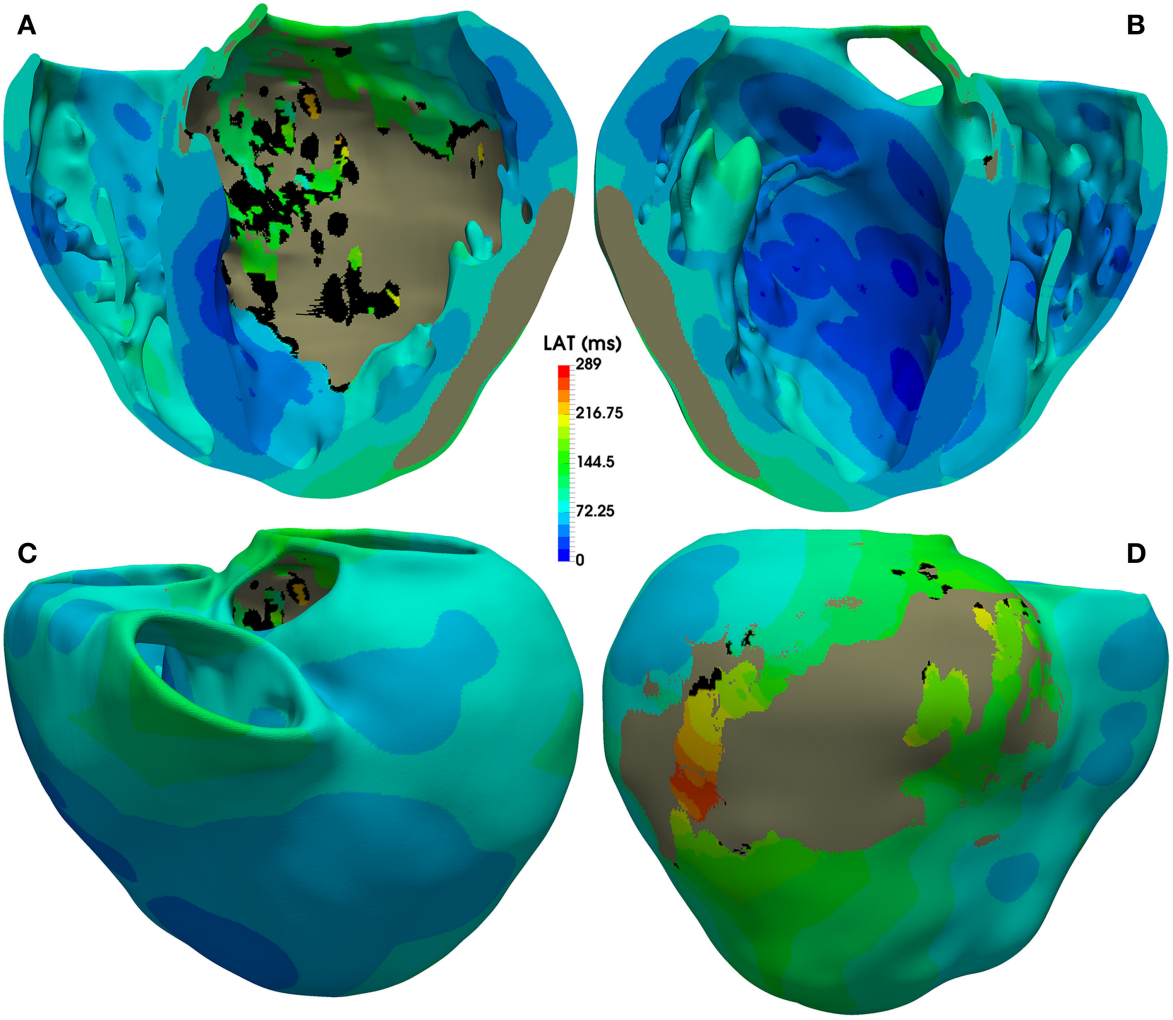
Activation map for CARTO-derived sinus activation, corresponding to the sixth heartbeat simulated with the ventricular model with ER and 10% fibrosis in BZ (model *#6*). Top row shows an anterior **(A)** and a posterior **(B)** view of a coronal cross-section (four-chamber plane) of the ventricular model, showing the activation on the endocardial surfaces. Bottom row also displays anterior **(C)** and posterior **(D)** views of the whole model, showing the activation at epicardial level. Black regions correspond to not activated tissue (not depolarized) due to fibrosis accumulation. Gray region represents the infarct scar, modeled as non-conducting tissue.

The activation map corresponding to sinus rhythm simulated with model *#6* (with ER and 10% fibrosis in BZ) is displayed Figure 7. As observed, most of the ventricular tissue is already activated in around 140 ms. However, certain regions of the BZ show a very late activation, especially the epicardial isthmus (see Figure 7D), where activation lasts up to 289 ms. Activation maps for this CARTO-derived sinus rhythm are very similar for all versions of the ventricular model. The only remarkable difference is the time that the tissue corresponding to the BZ takes to be fully activated, ranging from 243 ms for model *#1* (no ER and no fibrosis in BZ) to 296 ms for model *#8* (with ER and 30% fibrosis in BZ). Both factors slow down the activation of the BZ, with ER causing delays longer than fibrosis.

**Figure 8:**
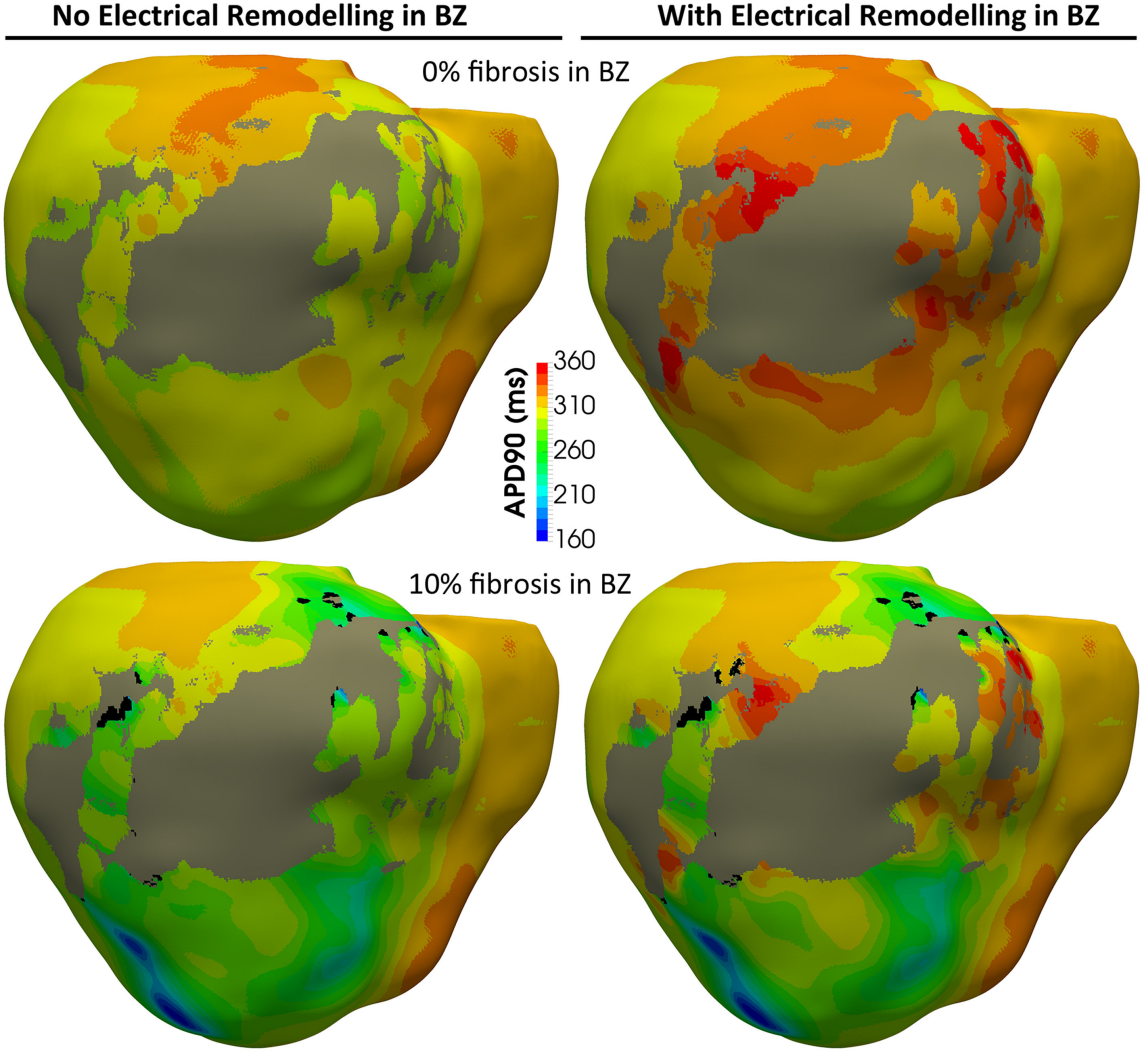
APD maps showing the epicardial surface of the posterior wall of the ventricular model, exhibiting the differences in repolarization patterns during simulated sinus rhythm for the four versions of the model resulting from the combination of absence and presence of ER with no fibrosis and with 10% image-based patchy fibrosis in the BZ. Those maps correspond to the sixth heartbeat simulated from the CARTO-derived activation pattern at a BCL of 800 ms (75 bpm). As observed, both the ER and the presence of patchy fibrosis in the BZ affects the APDs in the BZ, creating repolarization dispersion around the infarct scar. (see Supplementary Material for APD maps with 20 and 30% fibrosis in the BZ).

Figure 8 shows APD maps for four different versions of the ventricular model, depicting the repolarization dispersion (or APD heterogeneity) generated by both the ER and the presence of fibrosis in the BZ. The inclusion of ER in the BZ creates regions with longer APDs, while the presence of 10% image-based patchy fibrosis causes the opposite effect (APD shortening) in certain regions of the BZ. Thus, the combination of both factors increases the dispersion in the repolarization pattern, which may result in a higher arrhythmogenicity. For APD maps with 20% and 30% fibrosis in BZ, see Figure S2.

**Figure 9:**
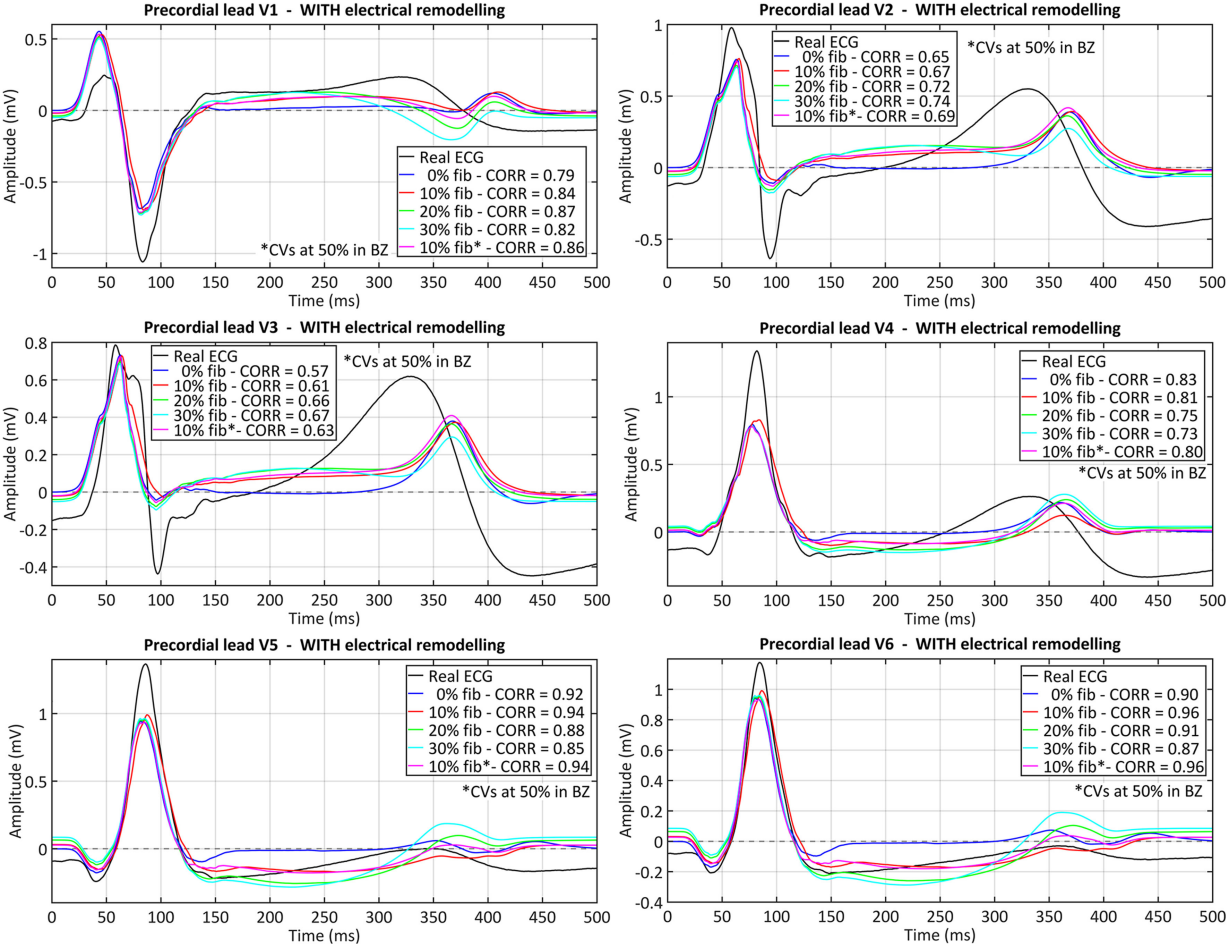
Comparison between real and simulated ECG signals recorded at precordial leads in sinus rhythm. These plots display the ECG simulated with all model versions including ER (models *#5–8*), combined with the four tested levels of image-based patchy fibrosis in the BZ. The ECG for the model version with ER, 10% fibrosis and the CVs reduced by 50% in BZ (10% fib*), instead of 75%, is also represented. Correlation coefficients (CORR) are included in the plots legend. Those signals were obtained by propagating through the 3D torso model the sixth heartbeat simulated from the CARTO-derived sinus activation pattern at a BCL of 800 ms (75 bpm). (see Supplementary Material for simulated ECGs with model versions without ER in the BZ).

The comparison between the real and simulated ECG (at the precordial leads) for all versions of the ventricular model including ER in the BZ is shown in Figure 9. The inclusion or absence of ER in BZ did not have an important impact on the simulated ECG in sinus rhythm (see Figure S3 for simulated ECGs with models without ER in BZ). Conversely, the presence of fibrosis in the BZ caused a deviation of the ST segment that matched that observed in the real ECG (see elevation in V1 and depression in V5 and V6, for instance). Furthermore, simulated QRS complex width and polarity was remarkably similar to the patient's one, with a signal correlation over 80%, except for V2 and V3 leads for which correlation was around 70%. The most important difference was the repolarization phase, since simulated ECGs showed a delayed T wave with respect to patient's one. Neither ER nor fibrosis in BZ appeared to have an important impact on repolarization, with only a slight effect on T wave magnitude depending on fibrosis level.

#### *In-silico* VT Induction

**Figure 10:**
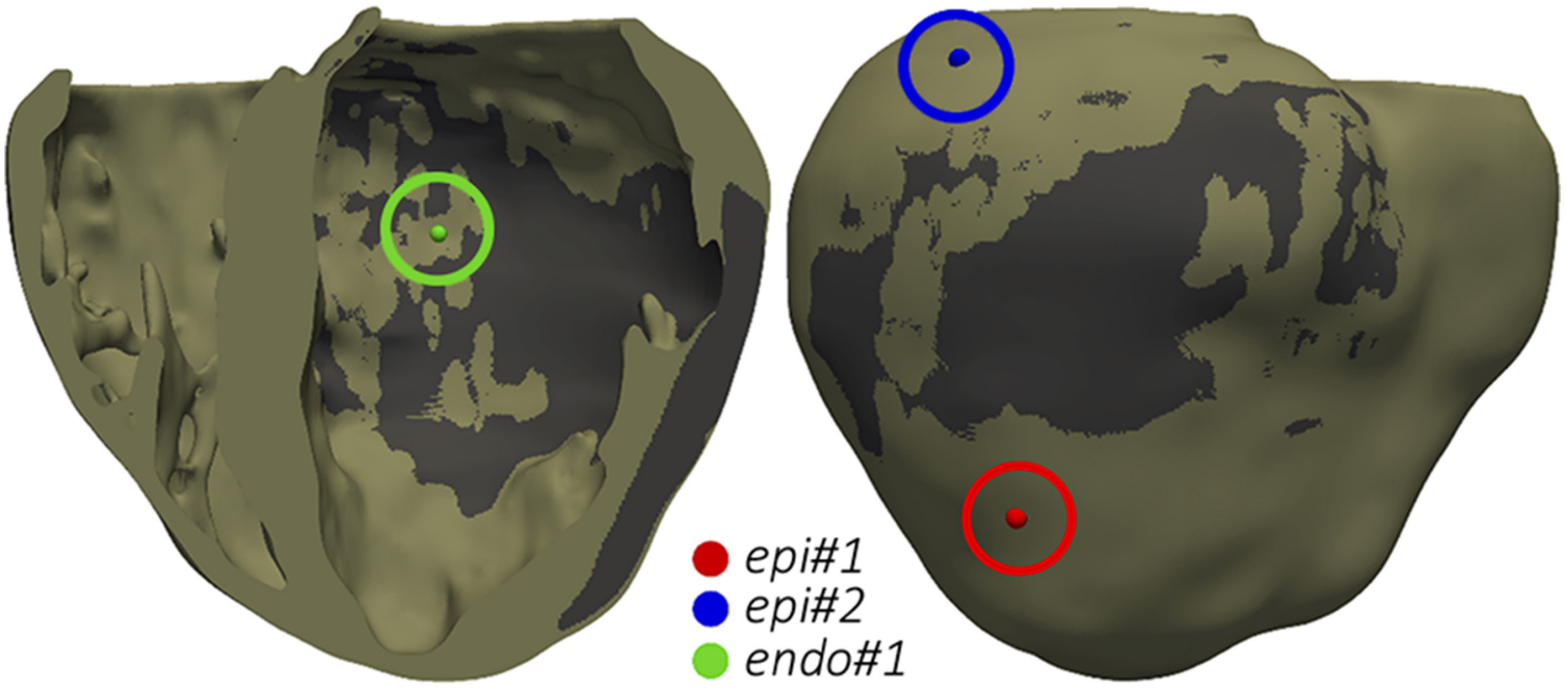
Pacing sites for the application of PES protocols aiming to test VT inducibility by computational simulation. The dark gray region represents the infarct scar.

The final goal of our simulation pipeline was to reproduce the clinical VT *in-silico* in order to study its mechanisms and to identify reentry circuits responsible for the VT as ablation targets. Among CARTO points projected onto the 3D model, we chose two locations tagged as pacing sites in the actual procedure and replicated the same programmed electrical stimulation (PES) protocol applied by the electrophysiologists in the EP laboratory. First tested pacing site (*endo#1*) was located on the LV endocardium (Figure 10, green sphere), while the second one (*epi#1*) was on the LV posterior epicardial wall below the apical side of the MI (Figure 10, red sphere). Aiming to explore the influence of pacing location, we included an additional point (*epi#2*) located on the LV epicardial posterior wall, over the basal side of the MI (Figure 10, blue sphere).

Starting from the steady-state after the stabilization in sinus rhythm, we paced the ventricles from each pacing site (one at a time) with a train of six stimuli delivered with a BCL of 600 ms (S1 phase), followed by a single stimulus (S2 phase) coupled at 400 ms after the last S1. If it failed to induce VT, we reduced the S2 coupling interval (CI) in steps of 10 ms until reaching positive VT induction or propagation block at pacing site. In the latter case, when VT was non-inducible by a single S2 stimulus, we repeated the PES protocol adding another premature stimulus (S3 phase) after the S2 phase, with both S2 and S3 stimuli coupled at the same CI. We followed that protocol for the eight model versions (Table 1) and the three pacing sites (Figure 10). Table 1 summarizes the results of all those *in-silico* tests of VT inducibility. Focusing on VT simulations at the organ level, such results show that our pipeline was able to replicate the outcomes of the VT inducibility tests performed in the EP laboratory. We achieved positive VT induction with several versions of the ventricular model from the two pacing sites (*endo#1* and *epi#1*) that succeeded in the real EP study. On the contrary, pacing site *epi#2* (not tested in the clinic) could not trigger VT on any model version.

**Figure 11:**
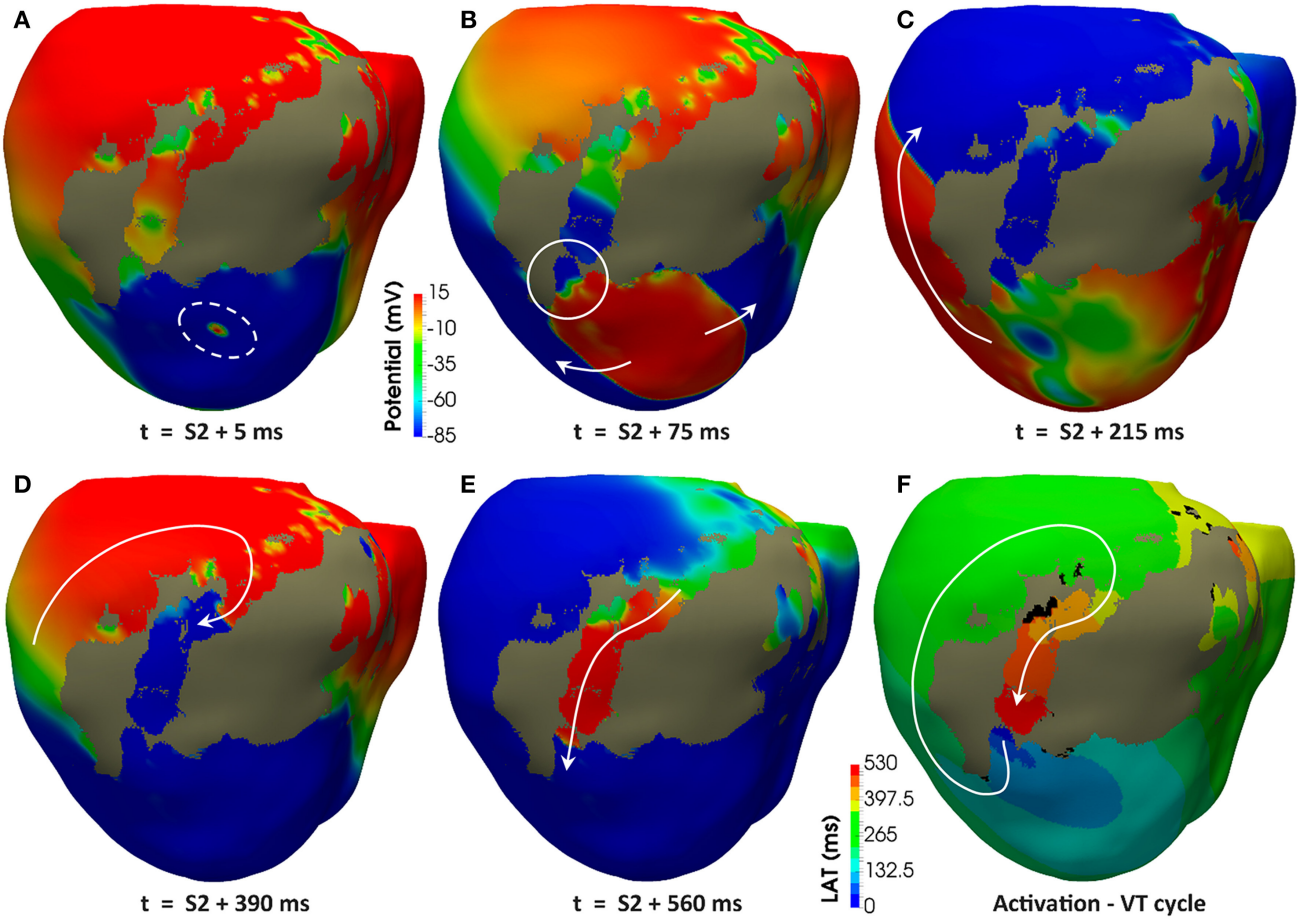
Reentrant monomorphic VT induced *in-silico* by PES protocol. Posterior views of the 3D ventricular model displaying potential maps at different time instants of the PES protocol from pacing site *epi#1* leading to positive VT induction on the model *#6* (ER and 10% fibrosis in BZ). Non-excitable scar is represented in gray color. White arrows indicate direction of the depolarization wavefront. **(A)** Only one premature stimulus (S2) was delivered at a CI of 360 ms. **(B)** The wavefront generated by S2 stimulus causes a functional propagation block at the lower side of the epicardial isthmus (see white circle). **(C)** The wavefront continues propagating around the infarct scar, but not through the epicardial isthmus due to the functional block caused by S2 stimulus. **(D)** The wavefront reaches the upper side of the epicardial isthmus after propagating all around the infarct scar. **(E)** The wavefront enters through the upper side of the epicardial isthmus, propagating across the channel until leaving it through its lower side (unidirectional block) to propagate again around the scar, giving rise to a reentrant activity leading to a self-sustained monomorphic VT. **(F)** Activation map of a cycle of the induced VT, confirming that the epicardial isthmus constitutes a SCC acting as structural substrate for this infarct-related VT.

Regardless the pacing site, all induced VTs showed a common mechanism characterized by a unidirectional block at the lower side (the most apical end) of the epicardial SCC previously described, consequently leading to reentry through its upper side (the most basal end). Figures 11A–E displays potential maps at different times, showing the propagation patterns generated by the S2 stimulus that gives rise to the unidirectional block and, subsequently, to the onset of the VT due to reentry. Such epicardial SCC enabled the perpetuation of the reentrant activity, showing a clockwise macroreentrant propagation pattern with the wavefront entering the SCC through its upper end and leaving it through the lower one (see Figure 11F), what triggered a self-sustained monomorphic VT with a BCL of 526 ms (114 bpm) on model *#6* (see Video S2).

In our model, the presence of ER in the BZ was necessary in three out of four configurations allowing positive VT induction (models *#5–7*). The only configuration providing positive VT induction without ER (model *#4*) required the highest fibrosis level (30%) in the BZ among tested ones, together with a CI for S2–S3 phases significantly shorter than that of cases including ER in BZ (see Table 1). Concerning pacing site *epi#2* that we added to those tested in the real EP study, the VT test was negative in all model versions, since the applied PES protocol never managed to induce unidirectional block at either of the ends of the epicardial SCC (see Video S3). Model *#8* was a special case, as it is the only configuration that always led to bidirectional block at the lower end of the epicardial SCC regardless both the pacing site and the CI for S2–S3 phases, blocking the propagation even in the S1 phase (see Video S4).

**Figure 12:**
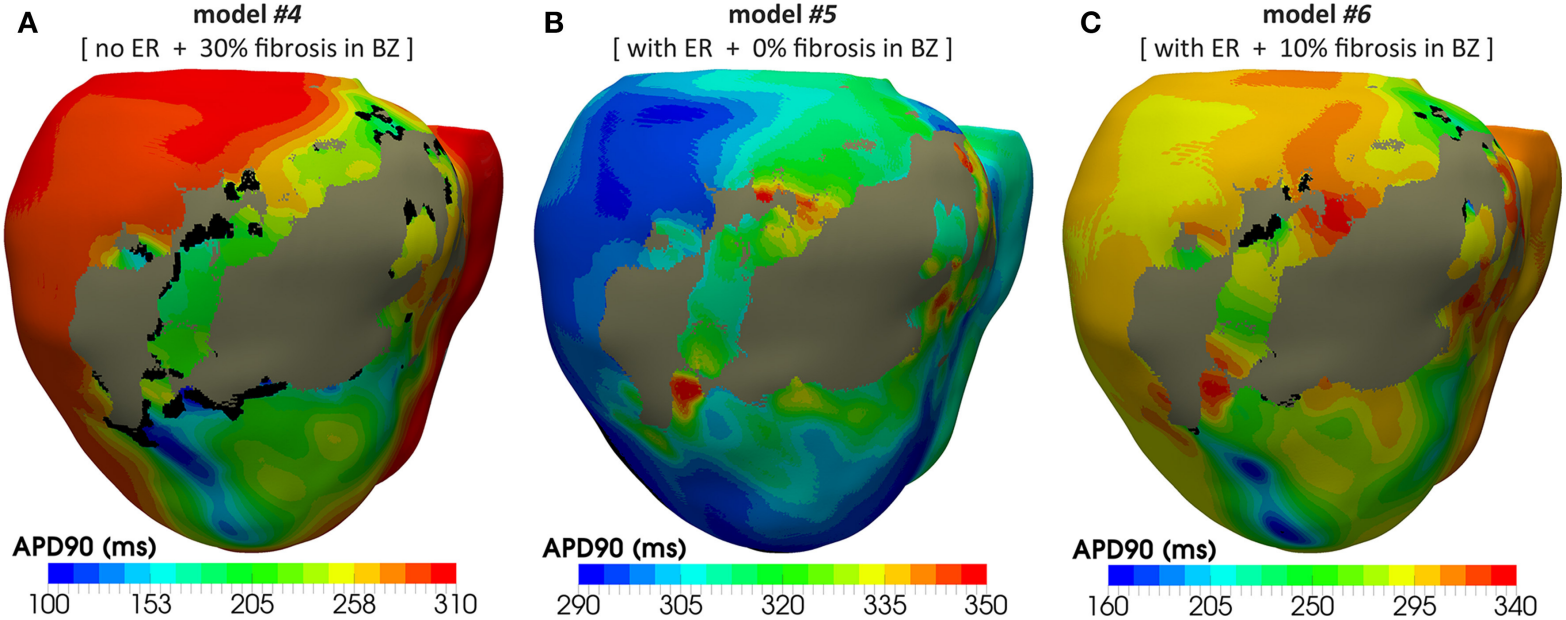
APD maps resulting from the propagation of the last stimulus of the S1 phase triggered from pacing point *epi#1*. These maps correspond to three out of the four different versions of the ventricular model that enabled positive VT induction in *in-silico* tests: **(A)** model *#4* (no ER and 30% fibrosis), **(B)** model *#5* (with ER and 0% fibrosis), and **(C)** model *#6* (with ER and 10% fibrosis). All of them show repolarization dispersion in the BZ surrounding the infarct scar, with a region of longer APDs at both ends of the epicardial SCC, especially at the lower side where the unidirectional propagation block that triggers the reentrant activity occurs. Black regions correspond to not activated tissue due to fibrosis accumulation.

Figure 12 shows APD maps for three different versions of the ventricular model, exhibiting considerable repolarization dispersion caused by either ER or fibrosis in the BZ, as already observed in sinus activation (see Figure 8 and Figure S2). Those maps, resulting from the propagation of the last stimulus of S1 phase, differ significantly between them, although sharing a common feature. All of them present regions of longer APDs (compared to the rest of BZ tissue) at both ends of the epicardial SCC that supports the reentrant activity. This effect seems more marked at the lower side of the SCC, where the unidirectional block that triggers the reentry occurs, especially in those models including ER in the BZ (Figures 12B,C). It is probably caused by a deeper impact of source-sink mismatches because of the narrower funnel shape of the lower end of the SCC compared to the upper one.

**Figure 13:**
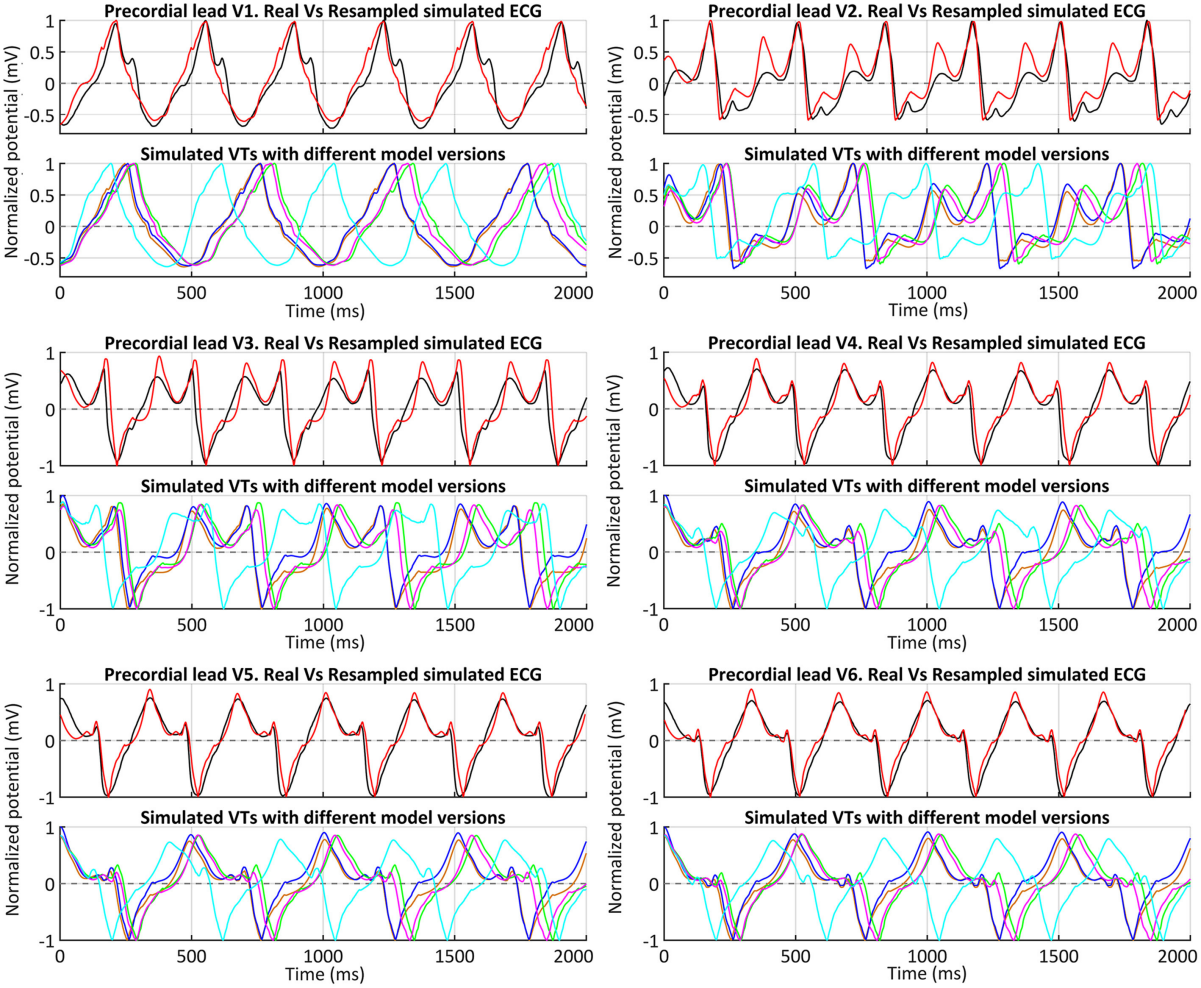
ECGs during clinical VT. Comparison between real (*black*) and simulated ECGs with different model versions for *in-silico* induced VT. Simulated ECGs correspond to all model versions that enabled a positive VT induction: models *#4* (*orange*), *#5* (*blue*), *#6* (*green*) and *#7* (*magenta*). The ECG for model *#6* (ER and 10% fibrosis in BZ) with the CVs reduced by 50% (instead of 75%) is also represented (*cyan*). Together with the patient's ECG (*black*), a resampled version of the ECG obtained with model *#6* and CVs reduced by 75% in BZ is displayed (*red*) to ease a visual comparison of the waveform of both signals.

Following the premise of only including non-invasive clinical data in our pipeline, we obtained the simulated ECGs for all successful VT induction using the torso model in order to assess the matching between the *in-silico* induced and the clinical VT by comparing both ECGs. The frequency of reentry around the scar was faster in the patient (BCL = 340 ms, 175 bpm) than in the simulations (BCL = 506–526 ms, 114–118 bpm) (see Table 1), probably due to differences in CVs mainly in the BZ including the SCC. It is noteworthy that CVs, as well as APDs, were not personalized but based on population data. Aiming to compare the morphology of precordial leads, we removed the frequency variability by resampling a simulated ECG and overlapped it to the patient's ECG. Figure 13 shows such comparison, where it is clearly appreciated that both morphologies present a high degree of correlation, with the simulated ECG following every signal deflection in the real signals in all precordial leads. Only lead V2 showed an enlarged amplitude in the simulated case in one of the sections of the signal. Hence, as in the case of sinus activation, V2 shows the most considerable differences with respect to the patient's ECG, along with V3 to a lesser extent. All configurations that managed to induce VT showed the same reentrant pattern depicted in Figure 11F, therefore resulting in highly similar ECGs, just revealing subtle differences in VT frequency, as observed in Figure 13 and detailed in Table 1.

After analyzing our simulation results, given the significant difference in frequency between clinical and simulated VT (175 vs. 115 bpm), we chose model *#6* and pacing site *epi#1* to perform some additional tests aiming to study the influence of different CVs in the BZ on VT features and its related mechanisms. With CVs reduced by 25% with respect to healthy tissue, the result for VT induction was negative, since unidirectional block never happened (see Video S5), reaching propagation fail at pacing site at CI of 290 ms. In contrast, the test with CVs reduced by 50% resulted in a positive VT induction, triggered again by unidirectional block at the lower side of the epicardial SCC, due to S2–S3 stimuli with 320 ms for CI. Such interval is notably shorter than the successful CI in the case of CVs reduced in 75% (see Table 1), in which VT furthermore resulted from a single S2 stimulus. Regarding the simulated ECG, the morphology of the monomorphic VT did not exhibit significant variations. However, the acceleration of the reentrant activity due to increased CVs in the BZ led to a considerably shorter BCL (425 ms, 141 bpm) (see Figure 13), closer to that of the clinical VT but still a 20% slower (175 vs. 141 bpm).

## Discussion

### 3D Modeling

In this study, we have built a detailed image-based 3D cardiac model that faithfully reproduces the *patient-specific* anatomy and function of the ventricles of a given subject (see Figure 1) from non-invasive clinical data, reaching a high level of anatomical detail. In contrast, fiber orientation and transmural EP heterogeneity were included based on population data. Unlike ours, most previous 3D ventricular models ignore papillary muscles and endocardial trabeculations, especially those based on *in-vivo* clinical images. Subtlety and high inter-subject variability of endocardial structures add difficulty to the already challenging task of segmenting *in-vivo* cardiac datasets. In our particular case, since the reentrant circuit is entirely confined at the epicardial level, neither papillary muscles nor trabeculations appear to have any impact on VT mechanisms. However, significant influence of some endocardial structures (e.g., moderator band in RV) on activation patterns has been observed both experimentally (Durrer et al., 1970) and by computational simulation (Bishop et al., 2010), as well as their potential role in the VT features (Kim et al., 1999b; Bogun et al., 2008; Walton et al., 2018). Further investigations with large cohorts of patients are required to assess the convenience of systematically including endocardial details in *patient-specific* models devoted to infarct-related VT simulations.

An essential element of our model was the anatomical and functional modeling of the infarcted region. We based our *patient-specific* anatomical MI model on cardiac DE-MRI (see Figure 2), which is the current gold-standard technique for *in-vivo* assessment of myocardial ischemic injury in clinical environments (Jamiel et al., 2017; Patel et al., 2017). Moreover, DE-MRI is a routine imaging technique recommended for infarcted patients referred for RFA procedures (Pedersen et al., 2014; Priori et al., 2015), so it satisfies our requisite of exclusively using non-invasive clinical data. DE-MRI enables the segmentation of the MI differentiating between infarct scar and BZ, that is crucial in order to study infarct-related VT mechanisms by computational simulation (Ringenberg et al., 2014; Ukwatta et al., 2016). Nonetheless, the proper definition of both types of tissue from *in-vivo* clinical DE-MRI images remains controversial (especially for the BZ), something that has given rise to a great variety of methods for this problem (Karim et al., 2016).

### Electrophysiological Modeling

Although it is not a completely acellular tissue (Sun et al., 2002; Rog-Zielinska et al., 2016), the infarct scar is mainly composed of extracellular matrix (collagen) (Cleutjens et al., 1999; van den Borne et al., 2010; Daskalopoulos et al., 2012). However, there is no representation for the extracellular space in the monodomain model. In such a case, a passive model (cellular level) combined with a low conductivity (tissue level) could be used to account for a slight electrotonic load caused by the scar (Ringenberg et al., 2014; Deng et al., 2015). We performed some initial tests assigning the MacCannell model to the scar region, as done in McDowell et al. (2011). When we computed the ECG, the signals showed considerable distortion caused by strong gradients resulting from the presence of such a large region (the scar occupies the 16% of LV in our model) that practically remained at resting potential across the simulations. This unwanted “side effect” would be critical for this work, since simulating the ECG properly is a key goal in order to compare our results to a real patient's recordings. Hence, we decided to disregard the potential electrotonic effect of the scar and modeled it as an electrical insulator by imposing no-flux boundary condition, as done in many other studies (Relan et al., 2011; Rantner et al., 2012; Arevalo et al., 2013, 2016; Ashikaga et al., 2013).

A few simulation studies focused on infarct-related VTs have also included fibrosis within the BZ. Some authors have randomly included fibrosis, either diffuse fibrosis modeled as an electrically passive tissue in a 3D model of rabbit ventricles (McDowell et al., 2011) or as micro-regions (patches) of non-conducting scarred tissue in a 3D canine model (Arevalo et al., 2013). Similarly, a very recent work included randomly generated patterns of non-conducting fibrotic tissue within the BZ in a model of infarcted rabbit ventricles (Campos et al., 2018). Another study faithfully reproduced fibrosis distribution in the BZ by means of highly detailed 3D models of wedge samples resulting from the reconstruction of high-resolution histological sections of infarcted rat hearts, considering it as non-conducting dense fibrotic tissue (Rutherford et al., 2012).

The gadolinium-based contrast agent used in DE-MRI is not specific for fibrosis, so the hyper-enhancement is assumed to be caused by its distribution through the extracellular space (Moon et al., 2004; Mewton et al., 2011), which is expanded in fibrotic regions resulting from the healing of MI (Hein and Schaper, 2001; Daskalopoulos et al., 2012; Seidel et al., 2016). Experimental observations based on histological sections have witnessed the presence of fibrosis infiltrated within the BZ (de Bakker et al., 1988; Smith et al., 1991; Rutherford et al., 2012; Tschabrunn et al., 2016), mainly showing the appearance of patchy/interstitial fibrosis. Importantly, this is considered the most arrhythmogenic kind of structural remodeling. Patchy fibrosis increases the susceptibility to unidirectional blocks due to source-sink mismatches and slows down conduction because of propagation delays caused by the tortuous pathways that the wavefront has to take, also leading to EGM fragmentation due to impaired activation (de Bakker et al., 2005; de Jong et al., 2011; Nguyen et al., 2014; Dhanjal et al., 2017). According to histological observations, it is widely assumed that voxels showing intermediate intensity levels in DE-MRI images, usually classified as BZ, correspond to a mixture of myocytes and fibrotic tissue (Hsu et al., 2006; Schuleri et al., 2009; Varga-Szemes et al., 2016). Consequently, we assumed that voxels in the BZ showing the highest intensity levels in the images probably correspond to pieces of myocardium having a high percentage of fibrotic tissue. Here, the intensity level of each DE-MRI voxel was mapped into a cluster of hexahedral elements in the volume mesh of ventricular model. This, along with the fact that the brightest voxels in the BZ are usually surrounded by others with similar intensities, makes our image-based method to generate patches within the BZ (see Figure 3), resulting in a pattern of patchy fibrosis similar to that observed in histological examinations (Schuleri et al., 2009; Rutherford et al., 2012). Finally, we defined image-based fibrosis up to 30% of the BZ (represented by the MacCannell model), since larger levels generated fibrotic barriers completely blocking the electrical propagation inside the epicardial SCC responsible for the VT, as for instance in the case of model *#8* described above. A recent study used a similar approach to include realistic fibrosis patterns based on DE-MRI images, although in that case the aim was to assess the influence of several strategies of fibrosis modeling on atrial fibrillation dynamics using a 3D model of human left atrium (Roney et al., 2016).

Regarding myocyte-fibroblast coupling, there is experimental evidence of the existence of this kind of heterocellular coupling via gap junctions in the remodeled myocardium resulting from MI (Camelliti et al., 2004; Schwab et al., 2013; Mahoney et al., 2016), as well as by tunneling nanotubes (Quinn et al., 2016). However, there is a lack of experimental studies focused on the characterization of myocyte-fibroblast coupling in the BZ of chronic MIs in terms of density and distribution of functional gap junctions connecting both types of cells (Ongstad and Kohl, 2016). Hence, magnitude and anisotropy of the electrotonic load caused by myocyte-fibroblast coupling in the BZ remain undetermined. Finally, we decided to consider this influence by means of a fibroblast model (MacCannell et al., 2007) and a conductivity value set for fibrotic tissue that mainly affects the myocyte-fibroblast coupling within the BZ by modulating the electrotonic interaction between both cell types.

Besides fibrosis, we also considered the ER in the viable but altered myocardium corresponding to the BZ that surrounds the infarct scar. However, it must be noted that all changes affecting ionic channels considered for the BZ are based on experiments with cells harvested from epicardial BZ of canine hearts (Pu and Boyden, 1997; Jiang et al., 2000; Dun et al., 2004). As recently discussed elsewhere (Connolly and Bishop, 2016), it is unclear to what extent such data are representative of the ER in BZ of human hearts. The presence of epicardial collateral circulation in canine hearts, not present in humans, is known to influence the formation of the epicardial BZ in dogs (Ursell et al., 1985; Schaper et al., 1988), which could give rise to functional differences with respect to the surviving myocytes in human BZ. On the other hand, all of those experimental studies (Pu and Boyden, 1997; Jiang et al., 2000; Dun et al., 2004) were performed a few days after the coronary occlusion, still in the healing phase of the MI rather than in its chronic stage (see Mendonca Costa et al., 2018 for a recent review). Nevertheless, these data have been widely used in previous studies (McDowell et al., 2011; Rantner et al., 2012; Arevalo et al., 2013; Ashikaga et al., 2013; Ringenberg et al., 2014; Deng et al., 2015; Hill et al., 2016) due to the lack of experimental data for ER in the BZ of chronically infarcted human hearts.

Our simulation results showed that VT inducibility was strongly related to slow conduction and APD heterogeneity in the BZ (including the SCC). Slow conduction in BZ is widely considered as a key factor in promoting reentry through SCC crossing the infarct scar, as it allows the working myocardium at the other side to recover its excitability before the arrival of the wavefront coming from the channel (Lazzara and Scherlag, 1984; de Bakker et al., 1993; Nguyen et al., 2014). However, the underlying mechanisms of this reduction in CVs still remain unclear, leading to a lack of consensus on the proper way to model it computationally. Some authors have reduced drastically only the transversal CV (McDowell et al., 2011; Deng et al., 2015). This approach could represent the severe reduction measured in gap junctional conductance in canine BZs only in the transverse direction (Yao et al., 2003) and/or the loss of side-to-side coupling between adjacent layers of myocytes due to the isolating effect of infiltrated fibrotic tissue (Spach and Boineau, 1997). Rutherford *et al*. managed to induce reentry in wedge models of infarcted myocardium due to the propagation delays exclusively caused by dense fibrosis (collagen) infiltrated within the BZ (Rutherford et al., 2012). Downregulation and lateralization of connexin 43 (i.e., gap junctions) have been also reported in the BZ (Smith et al., 1991; Severs et al., 2008). Downregulation of connexin 43 could result in an isotropic reduction of CV and its lateralization would exacerbate this effect in the longitudinal direction while keeping or even increasing transversal CV. Nonetheless, part of lateralized gap junctions of remodeled cardiomyocytes in the BZ are thought to be non-functional (Matsushita et al., 1999). Regarding tissue architecture, both DTI (diffusion tensor imaging) (Wu et al., 2006; Winklhofer et al., 2014) and histological preparations (Rutherford et al., 2012; Tschabrunn et al., 2016) have revealed fiber disarray within the BZ that consequently involves alterations in tissue anisotropy. Conversely, a recent study based on very high-resolution DTI observed good preservation of normal fiber orientation patterns in chronically infarcted regions of porcine and human hearts (Pashakhanloo et al., 2017). In conclusion, the slowed conduction in the BZ seems to derive from a complex combination of structural, electrical and gap junction remodeling that is not well-understood yet (de Bakker, 2017). In our case, aiming to reproduce macroscopically the global effect of all those combined factors affecting the propagation and promoting the reentrant activity, and in addition to the image-based patchy fibrosis, we imposed an isotropic reduction of CVs in the BZ as recently done by others (Ringenberg et al., 2014; Hill et al., 2016), testing reductions of 75, 50, and 25% for both longitudinal and transversal CVs with respect to the healthy myocardium.

### Sinus Rhythm Simulation

Regarding the personalization of cardiac activation, other authors have exploited EAMs to personalize electrical propagation in simplified models by adapting the so-called apparent conductivities (Chinchapatnam et al., 2008; Relan et al., 2011; Chen et al., 2016). Since we excluded invasively recorded data from our pipeline, we just benefited from CARTO data to validate the full 3D model (ventricles-torso set) in sinus rhythm by means of the simulated ECG resulting from the activation pattern provided by endocardial EAMs. The main limitations of EAMs are the lack of accurate automatic tools to fit the data to a 3D model and to annotate LATs, especially in regions showing pathological EGMs due to the presence of fibrosis and scarred tissue. The myocardium affected by the MI (scar and BZ) is the most thoroughly mapped region aiming to find SCCs across the scar as potential substrates for reentry (Soejima et al., 2002; Baldinger et al., 2016; Pokorney et al., 2016). However, in that region most of EGMs show low amplitude and/or very fragmented signals (Gardner et al., 1985; Bogun et al., 2005; Aliot et al., 2009). That is why we had to exclude such a large amount of CARTO points: 462 points removed out of 847 included in the original CARTO data. In this regard, new multi-array catheters, such as *PentaRay* for CARTO system (Biosense Webster, Inc., Diamond Bar, CA, USA) or *IntellaMap Orion* for Rhythmia system (Boston Scientific, Marlborough, MA, USA) (Mantziari et al., 2015), are currently helping to acquire less noisy and much denser maps. Despite current limitations, including the uncertainty in LAT annotations, our whole-body level approach allowed obtaining ECGs in sinus rhythm where precordial leads showed a good signal correlation with real ECGs (between 80 and 96% for V1, V4, V5, and V6), as well as a similar R-wave progression (see Figure 9). This, along with the good agreement in QRS complex duration, indicates that chosen values for CVs in healthy myocardium and for conductivities of organs and structures in torso model are within a proper range. Moreover, the coincidence in T wave polarity between real and simulated ECGs (except for V1) suggests an appropriate definition of transmural layers, since transmural heterogeneity is known to have a great influence on the repolarization phase in organ-level simulations (Okada et al., 2011; Perotti et al., 2015). As observed in Figure 9, simulated signals for V2 and V3 are the leads that show most notable differences compared to real ECG, not only in the repolarization phase but also in QRS complex, as real ECG shows a prominent negative S wave that simulations could not properly reproduce. This might be partially caused by the fact that in the EP laboratory a pad for defibrillation shocks is usually fixed on the left side of patient's chest, forcing the relocation of V2 and (sometimes) V3 electrodes away from their standard positions. Hence, since we placed the virtual electrodes for simulated ECG on the standard position of precordial leads (see Figure 5), this is likely to be an important error source specifically affecting V2 and V3. Moreover, considering that V2 and V3 were also the most different signals obtained from simulated VTs compared to the real ECG (see Figure 13), it seems highly probable that V2 and V3 electrodes were not placed in its standard positions in the EP laboratory. In conclusion, we consider this reproduction of the patient's ECG for sinus activation is an important step for the validation of our pipeline, even though it is usually omitted in other studies that also aim for planning of RFA interventions.

### *In-silico* VT Induction

The results of our simulation pipeline for *in-silico* tests of VT inducibility suggest that, in the presence of a SCC with slowed conduction, a key factor to cause a unidirectional block able to trigger a reentrant VT is the repolarization dispersion due to APD heterogeneity, as observed in Figure 12. In models *#5–7* such APD heterogeneity (see Figures 12B,C) comes from the increased APD associated with the ER in the BZ (see Figure 6), even causing permanent blocks when combined with high fibrosis levels (model *#8*). Nevertheless, in model *#4*, the APD heterogeneity responsible for the unidirectional block is exclusively caused by the presence of patchy fibrosis within the BZ, which by contrast gives rise to patches of tissue with decreased APD because of myocyte-fibroblast interactions (Figure 12A). This explains the need of a shorter CI to induce VT in model *#4* compared to those versions including ER in the BZ (see Table 1). On the other hand, the complete failure of pacing site *epi#2*, along with the fact that models *#4* and *#5* allowed positive VT induction from *epi#1* but not from *endo#1*, gives evidence of the important influence of the location of pacing sites on the result of VT inducibility tests and even on the morphology of induced VTs. If PES protocol delivered from *epi#2* had been able to cause a unidirectional block at the upper end of the epicardial SCC, for instance, the morphology of the resulting monomorphic VT would have been different. In such a case, macroreentry would show a counterclockwise propagation pattern that, consequently, would alter the morphology of the ECG during the VT episode. Nonetheless, not only the location of pacing sites but also the geometry of the SCC seems to have an essential role in VT mechanisms, what might be the reason for which in our model propagation blocks occur at the lower side of the SCC but never at the upper one. In our case, the lower end of the SCC is narrower than the upper (see Figure 11), which makes it more prone to functional propagation blocks due to source-sink mismatches caused by abrupt changes in the geometry of excitable tissue (Connolly et al., 2015; Ciaccio et al., 2018). With respect to the tests performed with different conductivities, simulation results confirm the great impact of CVs in the BZ both on the initiation mechanisms and on the frequency of infarct-related reentrant VTs.

Therefore, according to our simulation results, derived from a unique MI geometry, the most influential factors in promoting reentrant infarct-related VTs are the reduction of CVs and APD heterogeneity (repolarization dispersion) in the BZ, either caused by ER (model *#5*) or by the presence of fibrosis (model *#4*), as well as by the combination of both kinds of remodeling (structural and ER) (models *#6–7*). However, our results suggest that arrhythmogenicity is more strongly correlated with ER than with fibrosis in the BZ. Only in one case (model *#4* paced from point *epi#1*) we managed to induce VT in the absence of ER, requiring a high fibrosis level (30%) to create an APD heterogeneity enough to promote a unidirectional propagation block (Figure 12A), in combination with a shorter CI for premature stimulus. This is in agreement with that observed in Arevalo et al. (2013) and recently discussed in Trayanova et al. (2017), whose authors concluded that the presence of fibrosis in the BZ is not a necessary element in order to predict reentrant circuits by means of cardiac computational models, as long as ER in BZ is considered. Focusing on model versions including ER (models *#5–8*), moderate levels of patchy fibrosis in the BZ are thought to facilitate the initiation of VTs, as in the case of models *#6* (10%) and *#7* (20%) paced from point *endo#1* (see Video S6). However, elevated levels appear to prevent from VT mechanisms, as observed in model *#8* (30%), where the formation of a permanent bidirectional block avoids the self-sustained reentry and, consequently, the VT. This matches the conclusions reached in McDowell et al. (2011), where the authors observed an increased risk of infarct-related VTs for intermediate levels of diffuse fibrosis randomly distributed within the BZ, as well as a protective role against VT derived from higher levels. Hence, our approach of image-based patchy fibrosis seems to have a similar impact on infarct-related VT mechanisms.

Although the morphology of the signals of simulated ECG is highly similar to that of clinical VT (only V2 and V3 show notable differences, probably due to inaccurate electrode location) (see Figure 13), the BCL of *in-silico* induced reentry is larger than the patient's one, thus resulting in a slower VT. We think it is due to differences in CVs in the BZ and, consequently, in the epicardial SCC supporting the reentry, where the model is likely to conduct slower than the real case. When we increased CVs in the BZ up to 75% with respect to healthy tissue (i.e., a reduction of 25%), we could not induce VT because premature stimuli (S2–S3) stopped propagating at a CI too short to produce the functional block at the SCC entrance. The faster the propagation, the shorter the CI needed to generate a wavefront able to reach the SCC entrance within the time window in which its tissue is vulnerable to functional blocks. On the other hand, in the simulated ECGs in sinus rhythm we observed a delayed repolarization with respect to the patient's ECG (Figure 9), something that suggests that APDs in our model are larger than the patient's ones. We did not include the personalization of any parameter relative to cardiac EP in the design of our pipeline. However, we hypothesize that the choice of an ionic model with a basal APD shorter than ten Tusscher's one probably might allow inducing a reentry with a shorter CI for premature stimuli in spite of relatively high CVs in the BZ, triggering a reentry with a shorter BCL and, thus, a faster VT. Hence, we believe that including in our pipeline a coarse personalization of the APD based on the features of patient's ECG in sinus rhythm, as well as for CVs in healthy myocardium, could improve the performance of our approach. Although there are a few precedents in this regard (Relan et al., 2011; Chen et al., 2016; Gillette et al., 2018), further studies should be conducted in order to test such hypothesis.

As in this work, the ability to reproduce infarct-related VTs by means of image-based human cardiac computational models has been demonstrated in a number of previous studies (Relan et al., 2011; Ashikaga et al., 2013; Ringenberg et al., 2014; Arevalo et al., 2016; Chen et al., 2016; Deng et al., 2016; Prakosa et al., 2018). Concerning the global performance of our particular approach, we have been able to reproduce patient's ECG both in sinus rhythm and in clinical VT with good fidelity, considering that cardiac EP and myocardial architecture were not personalized. When PES protocols produce positive VT induction in the EP laboratory, electrophysiologists usually compare the resulting ECG to that registered during clinical VT episodes as a method to discern whether both VTs match or not, what helps in the process of choosing optimal ablation targets. Therefore, our pipeline completely based on non-invasive clinical data has the potential to replicate such process *in-silico*, such that it might become a helpful tool in therapy planning prior to RFA procedures aimed at finishing infarct-related VTs.

### Limitations

The major limitation of our work derives from the fact that it relies on only one case. Thus, this work must be considered as a proof-of-concept study that shows promising, yet preliminary results. Hence, further studies including a larger number of patients should be conducted in order to improve and validate our pipeline, as well as to strengthen our conclusions.

Some features of the 3D ventricular model were not personalized. Myocardial architecture, for instance, was generated by means of a rule-based approach based on population data. The only alternative is the use of DTI (Hsu et al., 1998; Scollan et al., 1998; Holmes et al., 2000), although *in-vivo* cardiac DTI remains highly challenging because of the artifacts caused by cardiac motion. However, some studies have compared simulation results performed on 3D ventricular models using rule-based methods and *ex-vivo* DTI (Bishop et al., 2009; Bayer et al., 2012), finding only minor differences in electrical patterns at global level, what confirms the validity and robustness of rule-based approaches for simulations of cardiac EP.

Currently, the electrical propagation through ventricular myocardium is known to be characterized by three distinct conductivities in longitudinal, transverse (within myocardial sheets) and normal (along transmural direction) axes (Hooks, 2007; Caldwell et al., 2009). Recent computational studies have shown the impact on propagation patterns that might result from considering such full electrical anisotropy, both in simulations at organ level in healthy ventricles (Johnston et al., 2016) and in simplified models of cardiac tissue under diseased conditions (Johnston et al., 2018). Instead, we considered a unique conductivity for all directions perpendicular to myocyte longitudinal axis, as it has been commonly assumed in most of the 3D computational studies of cardiac EP so far. Hence, the incorporation of full anisotropy to the cardiac EP modeling of our pipeline might be one of the future improvements, as well as the study of its influence on infarct-related VT inducibility.

We did not incorporate the cardiac conduction system in our ventricular model, although it was not expected to have a significant impact on the infarct-related VT mechanisms that we aimed to study in this work. Considering the longer APD of Purkinje cells and the delay at the Purkinje-myocardial junctions, the kind of VTs simulated in this study could not have been mediated by the Purkinje system. In any case, currently there is no *in-vivo* imaging modality capable of providing information about the *patient-specific* geometry of Purkinje network. There are a few recent studies proposing approaches to infer models of conduction system from endocardial EAMs (Vergara et al., 2014; Palamara et al., 2015; Barber et al., 2017), yet it would break our requisite of only using non-invasive clinical data collected prior to the RFA procedure. Then, the only alternative would be to include a synthetic Purkinje model. Nevertheless, in such a case it would be impossible to assess to what extent the impact on simulation results of that artificial Purkinje network (if any) would be faithfully replicating the influence of the patient's conduction system in such scenario.

The poor quality of the anatomical whole-torso MRI hampered the construction of a *patient-specific* torso model, forcing us to reuse and adapt an existing one. However, given the results yielded for simulated ECGs, it does not appear to have been an important drawback, at least in this particular case.

## Conclusions

Our 3D *patient-specific* model of the ventricles exclusively built from clinical data, in spite of avoiding the personalization of cardiac EP (based on population data), was able to reproduce the morphology of the clinical monomorphic VT suffered by the patient in the simulated ECGs. Furthermore, this allowed identification within the 3D model of the SCC responsible for the reentrant activity, matching the ablation target chosen by the experts in the RFA procedure that successfully eliminated VT inducibility in the patient. Hence, we have given a proof of the feasibility of developing 3D *patient-specific* computational models from clinical data aimed at simulation of the cardiac EP, with the potential to become a powerful tool for surgical planning. This approach could help to improve the currently low success rate of RFA procedures as well as to shorten surgery duration and, consequently, decrease the risk to the patient. It must be highlighted the great importance of the personalization of both cardiac anatomy and MI geometry from *in-vivo* high-resolution images to accurately locate reentry pathways as ablation targets by *in-silico* EP studies.

From the functional perspective, the computational modeling of the BZ remains a complex task due to the lack of experimental data from human hearts. In our study, the determinant aspects were the reduction of CVs and APD heterogeneity, which could be caused by ER in the BZ, a large amount of patchy fibrosis (30%) or a combination of both factors. The most arrhythmogenic versions of our ventricular model were those that combined the ER with intermediate fibrosis levels (10 and 20%) in the BZ. Such configurations generate an important repolarization dispersion (APD heterogeneity) around the infarct scar, especially in the vicinity of both terminal ends of the epicardial SCC, making the model more prone to the onset of reentrant activity due to functional unidirectional blocks in the channel.

Several challenging issues are still hampering the introduction of cardiac computational models as a common tool in clinical environments, such as the automatic and accurate reconstruction of *patient-specific* anatomy and infarct geometry from *in-vivo* images, especially from MRI modalities. Another important drawback is the current high cost of 3D computational simulations of cardiac EP at organ and body levels, whose computational burden demands the use of high-performance computational resources and long computing times.

## Ethics Statement

Regarding the ethical considerations, the protocol was approved by the Ethics Committee for Clinical Research of the Hospital Clinic Universitari de Valencia (Valencia, Spain), which certifies that the present study was conducted in accordance with the recommendations gathered in the Declaration of Helsinki, originally adopted by the General Assembly of the World Medical Association in 1964, and in its subsequent revisions. Furthermore, the patient, who underwent the standard clinical protocol, gave written informed consent for the use of his anonymized clinical data in this study.

## Author Contributions

AL-P, RS, MI, and JF jointly conceived this work and assessed the results. AL-P built the 3D models and performed the computational simulations for this work, all of this with the priceless help and close supervision from RS and JF. AL-P and RS wrote the manuscript. MI and RR collected and provided clinical data used in this work and gave valuable advice on the interpretation of electrophysiological data. MI checked the annotations of electrophysiological data and supervised this work, providing it with a clinical perspective. MB provided valuable advice on electrophysiological modeling issues, participated in the critical discussion of the results and contributed to the writing of the manuscript. All authors have read and approved the final version of the manuscript.

### Conflict of Interest Statement

The authors declare that the research was conducted in the absence of any commercial or financial relationships that could be construed as a potential conflict of interest.

## Figures and Tables

**Table:** 

**2D**	Two-dimensional
**3D**	Three-dimensional
**AP**	Action potential
**APD**	Action potential duration
**BCL**	Basic cycle length
**SCC**	Slow conducting channel
**DE-MRI**	Delayed enhancement-MRI
**EAM**	Electroanatomical map/mapping
**ECG**	Electrocardiogram
**EGM**	Electrogram
**FEM**	Finite-element method
**LAT**	Local activation time
**LV**	Left ventricle
**MI**	Myocardial infarction
**RFA**	Radiofrequency ablation
**RV**	Right ventricle
**SD**	Standard deviation
**VT**	Ventricular tachycardia
**DTI**	Diffusion tensor imaging
**EP**	Electrophysiology/electrophysiological
