# Supplementary material for: Personalized Cardiac Computational Models: From Clinical Data to Simulation of Infarct-Related Ventricular Tachycardia
Source: Front Physiol. 2019 May 15;10:580. doi: 10.3389/fphys.2019.00580 (PMC6531915; doi:10.3389/fphys.2019.00580)
Supplement: Supplementary file 7 [file Data_Sheet_1.pdf]

Supplementary Material

Personalized cardiac computational models: from clinical data to simulation of infarct-related ventricular tachycardia

Alejandro Lopez-Perez[1*], Rafael Sebastian[2], M. Izquierdo[3,4], Ricardo Ruiz[3,4], Martin Bishop[5], J.M. Ferrero[1]

[1] Center for Research and Innovation in Bioengineering (Ci2B), Universitat Politècnica de València, Valencia, Spain
[2] Computational Multiscale Simulation Lab (CoMMLab), Universitat de València, Valencia, Spain
[3] INCLIVA Health Research Institute, Valencia, Spain
[4] Arrhythmia Unit, Cardiology Department, Hospital Clínico Universitario de Valencia, Valencia, Spain
[5] Department of Biomedical Engineering, Division of Imaging Sciences & Biomedical Engineering, King's College London, London, United Kingdom

Supplementary Methods

Monodomain model for electrical propagation through cardiac tissue

The most biophysically realistic approach representing the reaction-diffusion process of electrical propagation in cardiac muscle is the so-called *bidomain model* (Geselowitz and Miller, 1983). Such model considers the cardiac tissue as an excitable continuum medium composed of two domains (intra- and extracellular) represented as volumetric conductors that coexist in the space, although separated by cell membranes. The mathematical formulation of bidomain model relates intra-, V_i, and extracellular, V_e, potentials by means of the two partial differential equations (PDE) written below.

$$\nabla \cdot (\boldsymbol{D_i} \cdot \nabla V_m) + \nabla \cdot (\boldsymbol{D_i} \cdot \nabla V_e) = C_m \frac{\partial V_m}{\partial t} + I_{ion}(V_m, v) \qquad \text{Eq. S1}$$

$$\nabla \cdot (\boldsymbol{D_i} \cdot \nabla V_m) + \nabla \cdot \big((\boldsymbol{D_i} + \boldsymbol{D_e}) \cdot \nabla V_e\big) = 0 \qquad \text{Eq. S2}$$

Where the transmembrane voltage, V_m, is defined as follows:

$$V_m = V_i - V_e \qquad \text{Eq. S3}$$

The right term of Eq. S1 represents the reaction phenomenon, where C_m corresponds to the electrical capacitance of cell membranes, as they are consider as dielectric barriers separating two conductive mediums (intra- and extracellular spaces), what matches the definition of an electrical capacitor. I_{ion} represents the ionic current flux through the cell membrane, which is described by the ionic model used at cellular level as a function of the transmembrane voltage, V_m, and a set of state variables (v) included in the model itself. In both PDEs (Eq. S1 and Eq. S2), $\boldsymbol{D_i}$ and $\boldsymbol{D_e}$ represent the volume-averaged conductivity tensors of intra- and extracellular spaces, respectively, as bidomain approach assumes different conductivities and anisotropy ratios for those two domains (Johnston, 2016; Roth, 1992).

The problem with bidomain model is the high computational burden associated with the challenging task of solving numerically an equation system consisting of a non-linear parabolic PDE (Eq. S1) coupled to an elliptic PDE (Eq. S2). The most popular alternative to overcome this drawback is the use of the *monodomain model* (Potse et al., 2006; Roth, 1988), which is a simplification derived from the bidomain approach. Such simplification comes from the assumption that variations of extracellular potential, V_e, are negligible, such that variations of transmembrane voltage, V_m, are mainly determined by the changes in the intracellular potential, V_i. Hence, the negligible impact of V_e on V_m allows uncoupling the two PDEs of bidomain model. Mathematically, those assumptions translate into considering that conductivity tensors of the two domains included in the bidomain model ($\boldsymbol{D_i}$ and $\boldsymbol{D_e}$) are parallel and with equal anisotropy ratios, so that they are proportional, i.e., related by a constant λ as shown in Eq. S4.

$$\boldsymbol{D_e} \;=\; \lambda \cdot \boldsymbol{D_i} \qquad \text{Eq. S4}$$

Using this expression (Eq. S4) to substitute $\boldsymbol{D_e}$ in Eq. S2 and combining the result with Eq. S1 in order to remove V_e, we obtain the following equation, which represents the monodomain model:

$$\nabla \cdot (\boldsymbol{D} \cdot \nabla V_m) \;=\; C_m \frac{\partial V_m}{\partial t} + I_{ion}(V_m, v) \qquad \text{Eq. S5}$$

Where $\boldsymbol{D}$ is the equivalent conductivity tensor of monodomain approach that is defined as follows:

$$\boldsymbol{D} \;=\; \frac{\lambda}{1+\lambda} \cdot \boldsymbol{D_i} \qquad \text{Eq. S6}$$

Then, the monodomain formulation describes the reaction-diffusion process of electrical propagation in cardiac muscle by means of a single parabolic PDE (see Eq. S5), whose right term represents the reactive part of the problem, while the left term corresponds to the diffusion phenomenon.

Approximation of the bidomain model for the simulation of the ECG

To obtain simulated ECG signals, we had to compute extracellular potentials, V_e, in ventricular myocardium and, then, compute the extracellular potentials generated by that electrical activity in the ventricles across the whole 3D torso model (from the heart up to body contour). However, extracellular space is missing in the monodomain model that we used at tissue level for the simulations with our 3D ventricular model. Therefore, to compute extracellular potentials we used an approximation of bidomain model, which was proposed by Keller *et al.* (Keller et al., 2010) and already used in previous works by our group (Ferrer-Albero et al., 2017; Martinez-Mateu et al., 2018). Such approximation of bidomain model comprises several steps, as explained below.

First, we interpolated the transmembrane potentials, V_m, computed for the ventricles domain (simulation at organ level), from the nodes of the hexahedral mesh of 3D ventricular model to those nodes of the tetrahedral mesh of 3D torso model labelled as ventricular myocardium. Note that transmembrane potentials in ventricles, V_m, were previously computed by simulation at organ level, using monodomain model by means of ELVIRA solver (Heidenreich et al., 2010). Then, combining the elliptic PDE of the bidomain model (Eq. S2) with the assumptions on conductivity tensors ($\boldsymbol{D_i}$ and $\boldsymbol{D_e}$) of monodomain approach (Eq. S4 and Eq. S6), we obtain the following expression that relates transmembrane voltage, V_m, and extracellular potentials, V_e, within the ventricles domain (Ω_H):

$$\nabla \cdot (\boldsymbol{D} \cdot \nabla V_e) = \frac{-1}{1+\lambda} \nabla \cdot (\boldsymbol{D} \cdot \nabla V_m) \quad in \quad \Omega_H \qquad \text{Eq. S7}$$

Where $\boldsymbol{D}$ is the equivalent conductivity tensor of monodomain model and λ is the constant of proportionality that relates conductivity tensors $\boldsymbol{D_i}$ and $\boldsymbol{D_e}$.

Then, to solve Eq. S7 in order to compute extracellular potentials, V_e, from interpolated transmembrane voltage, V_m, we considered that ventricles were isolated from the rest of torso model and immersed in a non-conducting bath, what results in Neumann boundary conditions on the interface between ventricles and surrounding medium ($\partial\Omega_H$) defined as follows:

$$\boldsymbol{n_H} \cdot (\boldsymbol{D} \cdot \nabla V_e) = 0 \quad on \quad \partial\Omega_H \qquad \text{Eq. S8}$$

Where $\boldsymbol{n_H}$ is the unit vector normal to the external surface of ventricles $\partial\Omega_H$ pointing outwards.

Once extracellular potentials in ventricles, V_e, were computed, then we considered the whole domain of 3D torso model (Ω_T) in order to compute such potentials from the ventricles up to the torso surface. Considering the torso as a solid conductor, we computed extracellular potentials in the torso domain by means of the following Laplace equation:

$$\nabla \cdot (\boldsymbol{D_T} \cdot \nabla V_T) = 0 \quad in \quad \Omega_T \qquad \text{Eq. S9}$$

Where V_T represents extracellular potentials within the domain of torso model (except for the ventricles) and $\boldsymbol{D_T}$ is the heterogeneous conductivity tensor of torso model defining its conduction properties. $\boldsymbol{D_T}$ was computed for every tetrahedral element of the volume mesh of 3D torso model using the different conductivities chosen for each kind of tissue (see conductivity values in (Ferrer et al., 2015)), considering isotropic conduction all over the torso except for the region of ventricular tissue, where we preserved the anisotropy previously set for cardiac muscle in the simulations at organ level.

For solving Eq. S9 in order to obtain extracellular potentials V_T all over the torso, we applied Dirichlet boundary conditions to force the continuity of extracellular potentials at ventricles-torso interface ($\partial\Omega_{HT}$), as shown in Eq. S10. In addition, we considered that the torso were surrounded by a non-conducting medium, thus imposing Neumann boundary conditions on the external surface of torso model ($\partial\Omega_T$), as defined in Eq. S11, where $\boldsymbol{n_T}$ is the unit vector normal to the torso surface pointing outwards.

$$V_e = V_T \quad on \quad \partial\Omega_{HT} \qquad \text{Eq. S10}$$

$$\boldsymbol{n_T} \cdot (\boldsymbol{D} \cdot \nabla V_T) = 0 \quad on \quad \partial\Omega_T \qquad \text{Eq. S11}$$

Finally, we addressed this problem by means of the FEM method, using the conjugate gradient method and incomplete Cholesky decomposition as a preconditioner for computing a numerical solution. In addition, we used a temporal resolution of 1 ms and the heterogeneous spatial resolution given by edge lengths of the tetrahedral volume mesh of torso model, as shown in Figure 5 (see in the main document of the article). To obtain simulated ECGs, we solved this problem by means of a custom MATLAB code.

References

Ferrer-Albero, A., Godoy, E. J., Lozano, M., Martínez-Mateu, L., Atienza, F., Saiz, J., et al. (2017). Non-invasive localization of atrial ectopic beats by using simulated body surface P-wave integral maps. *PLoS One* 12, e0181263. doi:10.1371/journal.pone.0181263.

Ferrer, A., Sebastián, R., Sánchez-Quintana, D., Rodríguez, J. F., Godoy, E. J., Martínez, L., et al. (2015). Detailed Anatomical and Electrophysiological Models of Human Atria and Torso for the Simulation of Atrial Activation. *PLoS One* 10, e0141573. doi:10.1371/journal.pone.0141573.

Geselowitz, D. B., and Miller, W. T. (1983). A bidomain model for anisotropic cardiac muscle. *Ann. Biomed. Eng.* 11, 191–206. doi:10.1007/BF02363286.

Heidenreich, E. A., Ferrero, J. M., Doblaré, M., and Rodríguez, J. F. (2010). Adaptive Macro Finite Elements for the Numerical Solution of Monodomain Equations in Cardiac Electrophysiology. *Ann. Biomed. Eng.* 38, 2331–2345. doi:10.1007/s10439-010-9997-2.

Johnston, B. M. (2016). Six Conductivity Values to Use in the Bidomain Model of Cardiac Tissue. *IEEE Trans. Biomed. Eng.* 63, 1525–1531. doi:10.1109/TBME.2015.2498144.

Keller, D. U. J., Weber, F. M., Seemann, G., and Dössel, O. (2010). Ranking the Influence of Tissue Conductivities on Forward-Calculated ECGs. *IEEE Trans. Biomed. Eng.* 57, 1568–1576. doi:10.1109/TBME.2010.2046485.

Martinez-Mateu, L., Romero, L., Ferrer-Albero, A., Sebastian, R., Rodríguez Matas, J. F., Jalife, J., et al. (2018). Factors affecting basket catheter detection of real and phantom rotors in the atria: A computational study. *PLOS Comput. Biol.* 14, e1006017. doi:10.1371/journal.pcbi.1006017.

Potse, M., Dube, B., Richer, J., Vinet, A., and Gulrajani, R. M. (2006). A Comparison of Monodomain and Bidomain Reaction-Diffusion Models for Action Potential Propagation in the Human Heart. *IEEE Trans. Biomed. Eng.* 53, 2425–2435. doi:10.1109/TBME.2006.880875.

Roth, B. J. (1988). The electrical potential produced by a strand of cardiac muscle: A bidomain analysis. *Ann. Biomed. Eng.* 16, 609–637. doi:10.1007/BF02368018.

Roth, B. J. (1992). How the anisotropy of the intracellular and extracellular conductivities influences stimulation of cardiac muscle. *J. Math. Biol.* 30, 633–646. doi:10.1007/BF00948895.

Supplementary Tables

	Feature	Single isolated cell: Healthy	Single isolated cell: Border Zone	Single isolated cell: Change	Cell embedded in a 3D tissue: Healthy	Cell embedded in a 3D tissue: Border Zone	Cell embedded in a 3D tissue: Change
Endocardium	Maximum amplitude	37.78 mV	23.75 mV	– 37.1%	25.03 mV	17.08 mV	– 31.7%
	Maximum upstroke vel.	92.66 mV/ms	70.17 mV/ms	– 24.3%	61.26 mV/ms	47.30 mV/ms	– 29.5%
	Average upstroke vel.	40.51 mV/ms	26.58 mV/ms	– 34.4%	20.62 mV/ms	17.90 mV/ms	– 13.2%
	APD at 90%	299 ms	398 ms	+ 33.1%	304 ms	404 ms	+ 32.9%
	Resting potential	– 85.23 mV	– 85.77 mV	– 0.64%	– 85.26 mV	– 85.78 mV	– 0.61%
M cells	Maximum amplitude	36.22 mV	21.50 mV	– 40.6%	25.20 mV	16.53 mV	– 34.4%
	Maximum upstroke vel.	92.27 mV/ms	71.15 mV/ms	– 22.9%	80.05 mV/ms	36.74 mV/ms	– 54.1%
	Average upstroke vel.	40.44 mV/ms	26.84 mV/ms	– 33.6%	26.66 mV/ms	14.48 mV/ms	– 45.7%
	APD at 90%	389 ms	491 ms	+ 26.2%	394 ms	494 ms	+ 25.4%
	Resting potential	– 85.10 mV	– 85.89 mV	– 0.94%	– 85.13 mV	– 85.91 mV	– 0.91%
Epicardium	Maximum amplitude	35.47 mV	20.47 mV	– 42.3%	23.86 mV	15.96 mV	– 33.1%
	Maximum upstroke vel.	91.80 mV/ms	71.01 mV/ms	– 22.6%	83.68 mV/ms	57.09 mV/ms	– 31.7%
	Average upstroke vel.	40.22 mV/ms	26.59 mV/ms	– 33.9%	26.08 mV/ms	16.47 mV/ms	– 36.8%
	APD at 90%	298 ms	393 ms	+ 31.9%	302 ms	394 ms	+ 30.5%
	Resting potential	– 85.18 mV	– 85.90 mV	– 0.84%	– 85.21 mV	– 85.91 mV	– 0.82%

Table S1. Changes in AP properties produced as a consequence of the modifications applied to the ten Tusscher model in order to include the electrical remodeling in the BZ, compared to the control version (not modified) used for healthy myocardium. This table shows measures resulting from a simulation with a single isolated cell (after stabilizing ionic models at a BCL of 800 ms) and for a cell embedded in a 3D tissue (i.e., surrounded by other cells) exclusively composed of remodeled myocytes.

Supplementary Figures

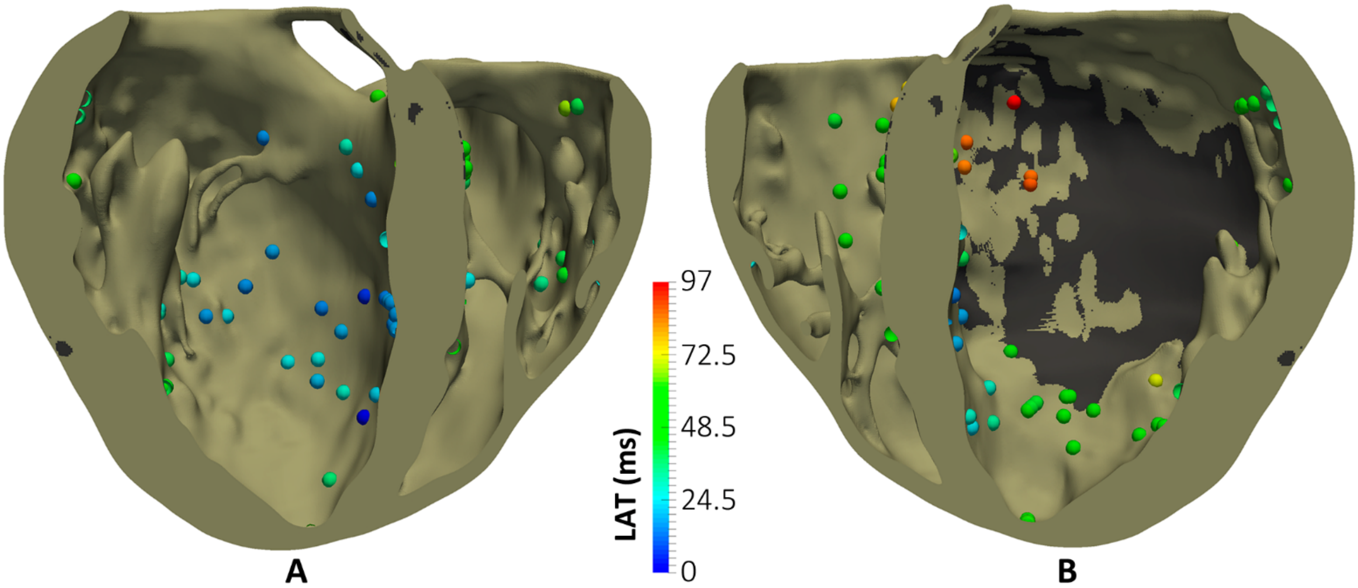

Figure S1. *Patient-specific* stimulation sequence derived from endocardial CARTO maps recorded in sinus rhythm during the real EP study. Posterior (*A*) and anterior (*B*) views of a coronal cross-section (four-chamber plane) of the 3D ventricular model, with infarct scar represented in *dark grey*. Spheres correspond to CARTO points projected onto LV (84 points) and RV (49 points) endocardial surfaces, with colour code representing LAT values for each point, which was measured from EGMs recorded via CARTO system. The earliest stimulated points (lowest LAT values according to CARTO EAMs) were located at mid-apical level on septal wall of LV endocardium (dark blue spheres in *A*), while the latest stimulated points were on the posterior wall (red and orange spheres in *B*) within the region surrounding the infarct scar, that is, in the BZ.

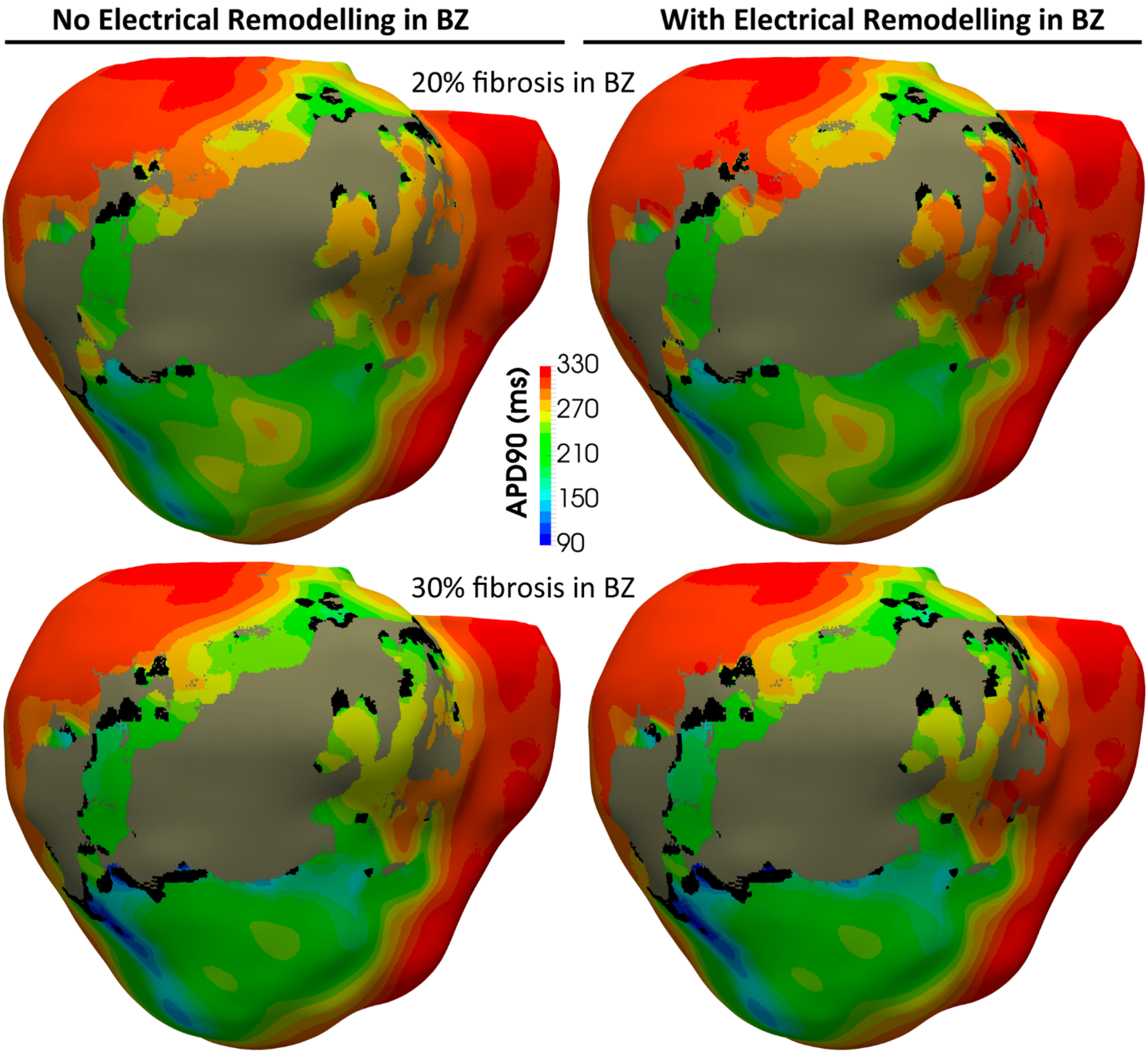

Figure S2. APD maps showing the epicardial surface of the posterior wall of ventricular model, exhibiting the differences in repolarization patterns during simulated sinus rhythm for the four versions of the model resulting from the combination of absence and presence of ER with two different levels of image-based patchy fibrosis in the BZ (20% and 30% fibrosis). Those maps correspond to the sixth heartbeat simulated from the CARTO-derived activation pattern at a BCL of 800 ms (75 bpm). As observed, both the ER and the presence of patchy fibrosis in the BZ affect the APDs in the BZ, creating repolarization dispersion around the infarct scar. The increase of the fibrosis level progressively shorten the APDs in certain regions of the BZ, also expanding the patches of not activated tissue (black regions) due to fibrosis accumulation.

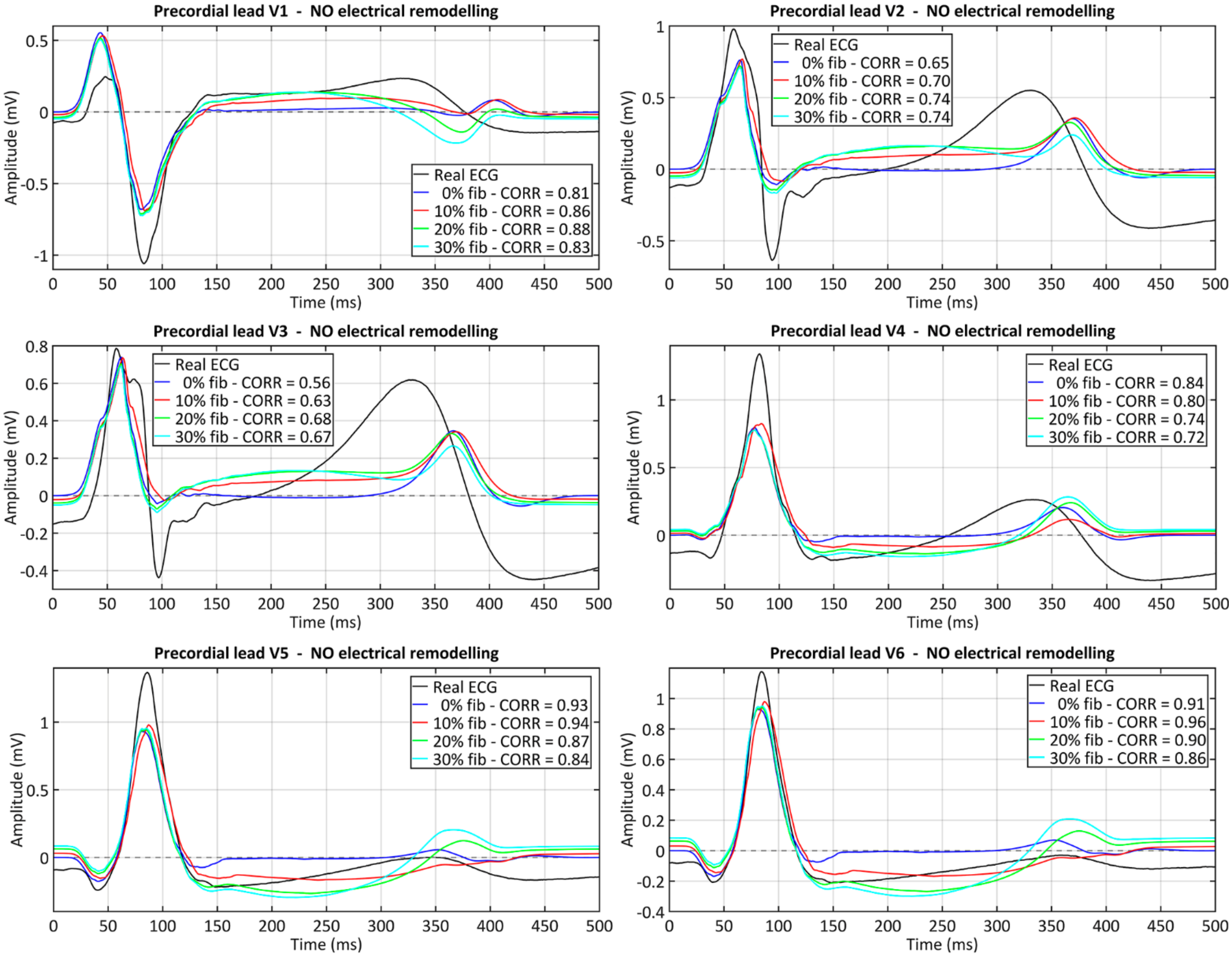

Figure S3. Comparison between real and simulated ECG signals recorded from precordial leads. These plots display the ECG simulated with all model versions without ER (models *#1-4*), combined with the four tested levels of image-based patchy fibrosis in the BZ. Correlation coefficients (CORR) are included in the plots legend. These signals were obtained by propagating through the 3D torso model the sixth heartbeat simulated from the CARTO-derived sinus activation pattern at a BCL of 800 ms (75 bpm).

Supplementary Videos

Video S1. Stabilization of myocyte-fibroblast coupling in model *#3* (no ER and 20% image-based patchy fibrosis in the BZ), with the CVs in the BZ reduced by 75% with respect to the healthy myocardium. On the left, the video shows an anterior view of a coronal cross-section (4-chamber plane) of the ventricular model, showing the endocardium of the LV posterior wall. On the right, there is a posterior view of the ventricular model, showing the epicardial surface. Note that the tissue corresponding to the infarct scar was removed in order to allow visualizing the process of myocyte-fibroblast coupling within the intramural layers. This video corresponds to a 1-second simulation with no stimulus applied on the model, showing the stabilization of the coupling between fibroblasts and adjacent myocytes in the BZ. To model fibrosis within the BZ at cellular level we used the MacCannell model of human ventricular fibroblast (MacCannell *et al.*, 2007). For viable myocardium in the BZ, in this case (model version with no electrical/ionic remodeling), we used the ten Tusscher model of human ventricular myocyte (ten Tusscher *et al.*, 2006). The significant difference in resting potentials between both models (around -49 mV for MacCannell's and -85 mV for ten Tusscher's) initially generates an ionic current flowing from the fibroblasts to the myocytes electrically coupled to them with intensity enough to trigger an AP in those myocytes. As clearly observed, this results in a multi-foci ectopic-like activation that propagates across the whole ventricular myocardium. After such initial spontaneous activation, the model reaches the steady state, since the fibroblast-myocyte couplings remains stable. Comparing the first frame of this video with the appearance of the model after the repolarization, one can appreciate a notable difference in the resting potentials in fibrotic regions (regions with elevated resting potentials) as a result of this stabilization process. At the beginning, there are abrupt transitions around the fibrotic patches, which turn into smoother potential gradients after the stabilization.

Video S2. Positive VT induction on model *#6* (with ER and 10% image-based patchy fibrosis in the BZ) paced from point *epi#1*, where the VT was triggered by a single premature stimulus (S2 phase) with a CI of 360 ms. In this simulation the CVs in the BZ were reduced by 75% with respect to the healthy myocardium. On the left, the video shows an anterior view of the ventricular model, showing the epicardial surface of a region not affected by the MI, as well as the four cardiac valves. On the right, there is a posterior view of the ventricular model, showing the epicardial surface of the region affected by the MI, where the grey region corresponds to the infarct scar, modeled as a non-excitable tissue (electrical insulator). This video shows the last S1 stimulus (t = 0 ms), followed by the single premature stimulus (S2 phase) (t = 360 ms). The wavefront resulting from the stimulus S2 produces a unidirectional propagation block at the lower (apical) end of the epicardial SCC (t = 440 ms), such that the wavefront travels around the scar and enters the SCC through its upper (basal) side (t = 700 ms). The wavefront propagates across the SCC and leaves it through its lower side (t = 900 ms), thus triggering a reentry that leads to a self-sustained monomorphic VT with a BCL of 526 ms (114 bpm). Moreover, one can observe a slight repolarization dispersion around the infarct scar caused by the electrotonic interaction between myocytes and fibroblasts due to the presence of 10% fibrotic tissue within the BZ.

Video S3. Negative VT induction on model *#5* (with ER and no fibrosis in the BZ) paced from point *epi#2* with a CI of 360 ms. In this simulation the CVs in the BZ were reduced by 75% with respect to the healthy myocardium. The video shows a posterior view of the ventricular model, exhibiting the epicardial surface of the region affected by the MI. Gray region corresponds to the infarct scar, modelled as a non-excitable tissue (electrical insulator). This video corresponds to a simulation with a length of 2 seconds. It shows the last S1 stimulus (t = 0 ms), followed by three premature stimuli (S2, S3 and even an extra S4 phase) at a CI of 360 ms delivered from pacing site epi#2. Neither S2 (t = 360 ms) nor S3 (t = 720 ms) phases give rise to a propagation block at the upper end of the epicardial SCC. Only applying an extra premature stimulus (S4 phase) and only for this configuration (model *#5*) we managed to create a functional propagation block at the upper end of the SCC. However, the S4-derived wavefront cannot enter the SCC through its lower end because it collides with another wavefront coming from the interior of the channel that was generated by the previous S3 stimulus. Consequently, that collision avoids the onset of a counterclockwise reentrant activity. On the other hand, due to the absence of patchy fibrosis within the BZ, the depolarization pattern does not show notable heterogeneities.

Video S4. Negative VT induction on model *#8* (with ER and 30% image-based patchy fibrosis in the BZ) paced from point *epi#1* with a CI of 360 ms. In this simulation the CVs in the BZ were reduced by 75% with respect to the healthy myocardium. On the left, the video shows an anterior view of the ventricular model, showing the epicardial surface of a region not affected by the MI, as well as the four cardiac valves. On the right, there is a posterior view of the ventricular model, showing the epicardial surface of the region affected by the MI, where the grey region corresponds to the infarct scar, modeled as a non-excitable tissue (electrical insulator). This video corresponds to a simulation with a length of 1 second. It shows the last S1 stimulus (t = 0 ms), followed by a single premature stimulus (S2) at a CI of 360 ms delivered from pacing site epi#1. As observed, the wavefront generated by the last S1 stimulus gives rise to a propagation block at the lower end of the epicardial SCC, such that it surrounds the infarct scar and enters the SCC through its upper side. That wavefront propagates across the SCC and reaches its lower end, where it seems to get blocked again, although it is uncertain due to the arrival of the wavefront generated by the stimulus S2 applied at t = 360 ms. The S2-derived wavefront surrounds the infarct scar and enters the epicardial SCC through its upper side as well, propagating again across the SCC. When it reaches the lower end of the SCC, the absence of a second premature stimulus allows to clearly appreciate how the wavefront gets blocked, confirming the presence of a permanent bidirectional block instead of a unidirectional functional block. Therefore, such bidirectional block at the lower side of the epicardial SCC avoid the onset of the sustained reentrant activity that would trigger an infarct-related VT. Moreover, one can observe an important repolarization dispersion around the infarct scar caused by the electrotonic interaction between myocytes and fibroblasts due to the presence of 30% fibrotic tissue (fibroblasts) within the BZ.

Video S5. Negative VT induction on model *#6* (with ER and 10% image-based patchy fibrosis in the BZ) paced from point *epi#1*. In this simulation the CVs in the BZ were reduced by 25% with respect to the healthy myocardium, so that none of the three premature stimulus applied (S2, S3 and even an extra S4 phase) could not induce the unidirectional propagation block that triggers the VT, in contrast to observed in Video S2 were CVs were reduced by 75%. On the left, the video shows an anterior view of the ventricular model, showing the epicardial surface of a region not affected by the MI, as well as the four cardiac valves. On the right, there is a posterior view of the ventricular model, showing the epicardial surface of the region affected by the MI, where the grey region corresponds to the infarct scar, modeled as a non-excitable tissue (electrical insulator). This video corresponds to a simulation with a length of 2 seconds. It shows the last S1 stimulus (t = 0 ms), followed by three premature stimuli (S2, S3 and S4 phases) at a CI of 310 ms delivered from pacing site epi#1. As it occurs in the case of every stimulus of S1 phase, the wavefront generated by each of the three premature stimuli manages to enter the epicardial SCC through its lower end. That wavefront propagates across the SCC and, at the same time, travels around the infarct scar, such that the wavefront leaving the SCC through its upper side collides with the one that has surrounded the infarct scar. Therefore, unidirectional propagation block never happens at any of the ends of the epicardial SCC, thus avoiding the onset of the reentrant activity that might trigger an infarct-related VT. Moreover, one can observe a slight repolarization dispersion around the infarct scar caused by the electrotonic interaction between myocytes and fibroblasts due to the presence of 10% fibrotic tissue within the BZ.

Video S6. Positive VT induction on model *#7* (with ER and 20% image-based patchy fibrosis in the BZ) paced from point *endo#1*, where the VT was triggered by the second premature stimulus (S3 phase) with a CI of 370 ms. In this simulation the CVs in the BZ were reduced by 75% with respect to the healthy myocardium. On the left, the video shows an anterior view of a coronal cross-section (4-chamber view) of the ventricular model, showing the endocardial surface of the posterior wall of the LV, which is affected by the MI. On the right, there is a posterior view of the ventricular model, showing the epicardial surface of the region affected by the MI. Grey region corresponds to the infarct scar, modelled as a non-excitable tissue (electrical insulator). At first, this video shows the last S1 stimulus (t = 0 ms). The wavefront resulting from the stimulus S2 (t = 370 ms) enters the epicardial SCC through its both ends (t = 720 ms), so that there is a collision in the interior of the channel (t = 805 ms). By contrast, the wavefront resulting from the stimulus S3 (t = 740 ms) produces a unidirectional propagation block at the lower (apical) end of the epicardial SCC (t = 1040 ms), while it manages to enter the SCC through its upper (basal) side. That wavefront continues propagating across the SCC until leaving it through its lower side (t = 1260 ms), thus triggering a reentry that leads to a self-sustained monomorphic VT with a BCL of 520 ms (115 bpm). Moreover, one can observe a considerable repolarization dispersion around the infarct scar caused by the electrotonic interaction between myocytes and fibroblasts due to the presence of 20% fibrotic tissue (fibroblasts) within the BZ.
